# Cultivable microbial diversity in speleothems using MALDI-TOF spectrometry and DNA sequencing from Krem Soitan, Krem Lawbah, Krem Mawpun, Khasi Hills, Meghalaya, India

**DOI:** 10.1007/s00203-022-02916-8

**Published:** 2022-07-17

**Authors:** Devender Mudgil, Dhiraj Paul, Sushmitha Baskar, Ramanathan Baskar, Yogesh S. Shouche

**Affiliations:** 1 Department of Environmental Science and Engineering, Guru Jambheshwar University of Science and Technology, Hisar, Haryana 125001 India; 2 National Centre for Microbial Resource, National Centre for Cell Science, Savitribai Phule Pune University Campus, Pune, 411021 India; 3 Environmental Studies, School of Interdisciplinary and Transdisciplinary Studies (SOITS), Indira Gandhi National Open University (IGNOU), New Delhi, 110068 India

**Keywords:** Cave, Microbial diversity, Geomicrobiology

## Abstract

The microbial diversity in the Indian caves is inadequately characterized. This study reports on the culturable microbial communities in caves from the Indian sub-continent. This study aims to expand the current understanding of bacterial diversity in the speleothems and wall deposits from Krem Soitan, Krem Lawbah, Krem Mawpun in Khasi Hills, Meghalaya, India. A culture-dependent approach was employed for elucidating the community structure in the caves using MALDI-TOF spectrometry and 16S rRNA gene sequencing. A high bacterial diversity and a greater bacterial taxonomic diversity is reported using MALDI-TOF spectrometry and 16S rRNA gene sequencing. High microbial enumerations were observed on dilute nutrient agar (5.3 × 10^3^ to 8.8 × 10^5^) followed by M9 minimal medium (4 × 10^4^ to 1.7 × 10^5^) and R2A medium (1.0 × 10^4^ to 5.7 × 10^5^). A total of 826 bacterial isolates were selected and preserved for the study. 295 bacterial isolates were identified using MALDI-TOF spectrometry and the isolates which showed no reliable peaks were further identified by 16S rRNA gene sequencing. A total 91% of the bacterial diversity was dominated by Proteobacteria (61%) and Actinobacteria (30%). In addition, bacterial phyla include Firmicutes (7.45%), Deinococcus-Thermus (0.33%) and Bacteroidetes (0.67%) were found in the samples. At the genus level, *Pseudomonas* (55%) and *Arthrobacter* (23%) were ubiquitous followed by *Acinetobacter, Bacillus, Brevundimonas, Deinococcus, Flavobacterium, Paenibacillus, Pseudarthrobacter*. Multivariate statistical analysis indicated that the bacterial genera formed separate clusters depending on the geochemical constituents in the spring waters suitable for their growth and metabolism. To the best of our knowledge, there are no previous geomicrobiological investigations in these caves and this study is a pioneering culture dependent study of the microbial community with many cultured isolates.

**Supplementary Information:**

The online version contains supplementary material available at 10.1007/s00203-022-02916-8.

## Introduction

Caves are geo-biologically interesting ecosystems and are usually characterized by speleothems, slimy wall deposits and biofilms that are known to host diverse microbial communities. These habitats are considered as extreme environments. Most microbial communities in caves rely on oligotrophic or chemotrophic modes of nutrition (Barton et al. 2007). These unique habitats need to be explored for microbial diversity studies as they are sites for the identification of novel microbes, which can have potential applications such as in the production of antibiotics, in bioremediation of contaminated sites, understanding microbe-mineral interactions and in the search for life on other planets (Boston et al. 2001). For example, *Actinobacteria*, identified from caves may produce novel bioactive compounds (Ghosh et al. 2017).

Most deep cave passages are totally devoid of light that limits primary production. Therefore, understanding of life in dark oligotrophic cave environments helps to delineate the mechanisms of metabolic flexibility of microorganisms that do not depend on sunlight for their metabolism.

Microbes have been reported in secondary mineral deposits in caves such as stalagmites, stalactites, sulphur compounds, and oxides of iron and manganese (Northup et al. 1997). Most scientists have based their evidences on microscopic studies, geochemical observations, culture-dependent microbiology, and molecular phylogenetic studies for understanding microbe-mineral processes in caves. Recent research has also suggested microbial links to the different minerals and fibers observed in caves (Baskar et al. 2016; Maciejewska et al. 2017). Scientists have also worked on the importance of such diverse microbial communities and their roles in cave biomineralization processes. Some examples include that of microbial biofilms, ferromanganese deposits and wall deposits in caves (Barton et al. 2014). Studies relating to cave microbial diversity have also been reported in Lechuguilla Cave, New Mexico (Northup et al. 2003); Herrenberg, Germany (Rusznyák et al., 2012); caves from Venezuela (Barton et al. 2014); Tjuv-Ante’s Cave, Sweden (Mendoza et al. 2016); Heshang caves (Yun et al. 2016a,b; Zhao et al. 2018); and Lava caves, USA (Lavoie et al. 2017).

Molecular techniques for the characterization of cave microbiomes has helped in advancing our knowledge of cave ecosystem community structures and their functions (Ortiz et al. 2013). Studies on microbial communities in caves have mostly focused on dripping waters (Marques et al. 2019), cave sediments (Adetutu et al. 2012), cave wall surfaces (Ortiz et al. 2013) and biofilms (Jones et al. 2012, 2014). Researchers have also studied how factors such as pH (Yun et al. 2016a), nutrition (Cloutier et al. 2017), and trace elements (Wu et al. 2015). can shape microbial communities in caves.

Microbial diversity studies in caves reveal the diverse microbial groups and communities residing in the energetically different parts of the cave. Several microbial groups have been identified in caves such as: Proteobacteria, Actinobacteria, Firmicutes, Acidobacteria, Verrucomicrobia, Planctomycetes, Nitrospirae, and Bacteroidetes (Barton and Jurado 2007; Tomczyk-Żak and Zielenkiewicz 2016). These diverse microbes thrive on the organic/inorganic constituents and gases present in the cave and speleothems.

Presently, geomicrobiologists use two broad techniques for analysing the diversity in speleothems. These include: (1) culture-based and (2) molecular phylogenetic analysis (Barton et al. 2001). Culture-based studies have limitations for microbial identification procedures because of the specific nature of nutrients required for specific microbes. Using this method, < 1% of all microorganisms in an environment can be cultivated (Amann et al. 1995). This limitation can be overcome by applying molecular phylogenetic techniques. However, Donachie et al. (2007) compared both culture-dependent and culture-independent methods and advocated that culture-dependent techniques are equally important. Merely relying on the ribosomal approach overlooks a significant fraction of phylogenetic diversity easily determined by cultivation methods which leads to significant gaps in microbial community diversity data. To capture the full range of microbial diversity in a community we need to implement broad strategies that employ both culture and molecular approaches. To understand and decipher the role of microorganisms in the Indian cave ecosystems, a multidisciplinary approach was followed.

In an earlier report by our team, microbes were isolated from the same caves and tested in vitro for microbe-mineral precipitation experiments (Mudgil et al. 2018). Many of the isolated strains in our study showed biomineralization potentials. Cultivation is an important part of the description of a microbial community as the cultivated rare taxa represent a reservoir of biological diversity that is seldom retrieved in molecular studies.

The present study reports on the culturable microbial diversity in speleothems and cave wall deposits from the same caves using MALDI-TOF spectrometry (Matrix-assisted laser desorption-ionization time of flight mass spectrometry) (Rahi and Vaishampayan 2020) and 16S rRNA gene-based sequencing. MALDI-TOF was used for identification of all the 826 strains isolated. For those isolates that did not show reliable peaks in the MALDI-TOF based identification; the genomic DNA was extracted and further identified by 16S rRNA gene-based sequencing analysis. The study also aimed to understand the link between the bacterial heterogeneity in the caves and geochemical parameters. The studies were performed for documenting the microbial diversity in caves and in analysing specific phylogenetic groups involved in cave bio-mineralization processes.

## Materials and methodology

### Speleological setting

India is home to many unexplored caves. For the present study, samples were collected from the East Khasi Hills (total area 2748 sq. km; Latitude: 25° 36′ 82ʹʹ N, Longitude: 91°75′39" E) of Meghalaya. The Mawsynram district is the wettest place with an annual average rainfall of about 11, 872 mm. For the study, three caves situated near Mawlyngbna village (25° 14′ 10.7ʹʹ N, 91° 33′ 37.0ʹʹ E) namely, Krem Lawbah, Krem Soitan and Krem Mawpun were sampled (Supplementary Fig. 1a, b).

#### Krem Mawpun

This cave is located in the Mawsynram-Mawlongbna region. It was partially explored in the year 2010 and extends from a depth of 1694 to 2541. This cave is India’s second longest sandstone cave known to date. In this cave, there were many small soda-straw structures hanging from the ceiling of the cave with drip waters dripping from the straws and stalactites (Supplementary Fig. 2a–c). These soda-straw structures were sampled for the study. At many places inside the cave, the water level was low, and small pigment-less millipedes were observed. Some aquatic animal species were also observed swimming actively in small pools with low water levels.

#### Krem Lawbah

This cave is also known as “Krem Pamskei” and is located 16 km south of Mawsynram (the wettest place on the earth). Meghalaya adventure association (MAA) have explored this cave up to the depth of 1189 m. Beyond a few metres of the cave entrance, the cave was completely dark. Within the cave, passages were extremely narrow, necessitating crawling through the small openings and passages to reach the larger chambers. Krem Lawbah hosts stalactites, columns and flowstones (Supplementary Fig. 2 h–j). Inside the cave, there were numerous drip-water points with a slow drip-rate. Bats, spiders, and hyphae-like structures adhered to the rocks were also discovered in the cave. All the samples were unaffected by any anthropogenic activity.

#### Krem Soitan

This cave is also known as Devil’s cave as ‘Soitan’ in the native language means ‘devil’. It is located south of Mawsynram at the base of a cliff near a river, close to the Bangladesh border. The cave entrance measured approximately 2 m in length. There are photic, twilight, and aphotic zones in the cave. At the intersection of the photic and twilight zones, a pool of clear water was observed. The cave is home to magnificent mineral formations such as stalactites, stalagmites, columns, draperies, and moonmilk, as well as spectacular mushroom-like structures (Supplementary Fig. 2d–g). Additionally, bats and spiders were spotted inside the cave.

### Sample collection

Samples (up to 10–20 g) were collected *in-situ* using sterilized forceps, scalpel, geologic hammer and chisel from areas that were minimally contaminated (undisturbed by any human/anthropogenic activities). All the samples were present in a remarkable state of preservation without any antropogenic or geologic disformity. Sterile geologic hammer was used to chip off the speleothems samples and wall deposits were scraped using sterile scalpel. Samples were then placed into sterilized zip lock sachets and bottles using aseptic techniques. Geologic hammer and scalpel were sterilized using ethanol after every sample collection. Fresh samples (pool and drip waters) were collected in an icebox in 250-ml sterile polypropylene bottles. Portable instruments were used to determine the pH, conductivity, humidity, and temperature in situ.

Seventeen speleothem samples [one from Krem Mawpun (Supplementary Figs. 2a–c), nine from Krem Soitan (Supplementary Figs. 2d–g) and seven from Krem Lawbah (Supplementary Fig. 2 h–j)] were collected from minimal-contaminated areas. The samples also included four wall deposits from Krem Lawbah and one moonmilk from Krem Soitan. All the samples were collected from aphotic zones extending from 89–289 m in Krem Soitan and 125–850 m in Krem Lawbah throughout the length of the caves. In Krem Mawpun, only one sample was collected at about 300 m from the cave entrance (Supplementary Table 1). This was because the length of Krem Mawpun was small when compared to the other two caves and the areas inside the cave were quite difficult to access. Some samples were hard, and some had a little moisture. The samples were predominantly brown and the moonmilk had a whitish tinge (Supplementary Fig. 2f). Further, 16 water samples were collected from the 3 caves (Supplementary Table 1). The samples were transported in an icebox to the Environmental Geology Laboratory, Department of Environmental Science and Engineering, Guru Jambheshwar University of Science and Technology, Hisar and National Centre for Microbial Resource, Pune. They were immediately processed and stored at − 4 °C and − 20 °C, respectively, for a variety of geochemical and microbiological studies (experiments for microbiology were conducted within a week of sample collection). Speleothems (stalactite, stalagmite), moon milk, and wall deposits (1 g) were powdered aseptically in a sterilized mortar and pestle inside the laminar hood, pestle and mortar was cleaned after every sample grind.

### Geochemistry/mineralogy

#### Spring water chemistry

The electrical conductivity, pH, total dissolved solids, and salts of the spring waters were measured on the spot in the caves using handy multiparameter instrument (EUTECH Instruments PCSTESTR^™^ 35). Sulphate, phosphate, nitrate, chloride, acidity, alkalinity, total hardness, calcium, magnesium, carbonate and bicarbonate were determined using standard procedures (APHA 2005). Sodium and potassium were measured using a flame photometer. Total organic carbon, inorganic carbon and total carbon content in the water samples were determined using TOC-L (Shimadzu) at the Department of Environmental Science Laboratory, Guru Jambheshwar University of Science and Technology, Hisar, India.

### Microbiological analyses

#### Culture-dependent studies

##### Isolation and enumeration of the cultivable isolates

The culturable, aerobic, heterotrophic bacteria of the total microbial community were isolated from the speleothems (Table 1). The microbes were enumerated by dilution plate technique using three different media namely diluted (1:100) nutrient agar (Hi-media; Beef extract 0.03%, Peptone 0.05% and Agar 1.5%), M9 minimal salts (M9 salt solution (10X)- Na_2_HPO_4_, KH_2_PO_4_, NaCl, NH_4_Cl; glucose (20%) (w/v) as a carbon source; MgSO_4_, CaCl_2_, biotin, thiamine, and trace elements (Atlas 1993) and R2A agar medium for the isolation of oligotrophic bacteria (Reasoner and Geldreich, 1985). One gram of the powdered speleothem samples was mixed with 9 mL of 0.9% saline and serially diluted (10^–1^ to 10^–5^) in normal saline. 100 μL of suspension of each dilution was cultivated on respective agar plates in triplicate and incubated at 23 °C for a month (Baskar et al. 2016). Throughout the incubation period, the numbers of bacterial colony forming units (CFUs) were counted every 24 h. Morphologically distinct colonies were selected and purified by repeated (4–5 times) sub-culturing on the respective media. After purification, the purified bacterial strains were preserved at − 80 °C with 15% glycerol.

**Table 1:** Viable cell counts of bacteria cells in different media from speleothems samples collected from Khasi hills caves, Meghalaya

Media	Media reference	Used in detection of microbes	Carbon/nutrient source in the medium	KSSMc1	KSSTc1	KSSTc2	KSSTc3	KSSTc4	KSSTc5	KSSTc6	KSSTc7	KSSTc8	MPSTc1	LBSTc1	LBSTc2	LBSTc3	LBWDc1	LBWDc2	LBWDc3	LBWDc4
Dilute nutrient agar	Nutrient agar (NA) Hi-media	Heterotrophs, non-fastidious organisms	Glucose, peptone, yeast, beef extract	7.2 × 10^4^	NG	5.3 × 10^3^	3.7 × 10^5^	3.2 × 10^4^	8.8 × 10^5^	NG	7.1 × 10^4^	6.4 × 10^4^	4.0 × 10^4^	NG	NG	6.0 × 10^4^	NG	4.9 × 10^4^	4.7 × 10^4^	8.5 × 10^4^
R2A agar	R2A agar (Reasoner and Geldreich 1985)	Oligotrophic bacteria	Casein hydrolysate, Peptone, yeast extract, magnesium sulfate, sodium pyruvate	5.78 × 10^5^	1.0 × 10^4^	5.50 × 10^5^	5.6 × 10^5^	4.54 × 10^4^	7.86 × 10^4^	NG	6.77 × 10^4^	5.99 × 10^4^	3.47 × 10^4^	1.2 × 10^4^	1.7 × 10^5^	4.86 × 10^4^	2.48 × 10^4^	5.6 × 10^5^	4.52 × 10^4^	7.6 × 10^4^
M9 Media	Minimal Media (M9 salts) (Atlas 1993)	Heterotrophs; not auxotrophs	Na_2_HPO_4_.7H_2_O; KH_2_PO_4_; NaCl; NH_4_Cl; MgSO_4_; glucose	8.3 × 10^4^	NG	6.2 × 10^4^	8.3 × 10^4^	1.5 × 10^5^	4 × 10^4^	NG	4.5 × 10^4^	4.6 × 10^4^	NG	NG	NG	7.1 × 10^4^	NG	1.7 × 10^5^	5.1 × 10^4^	NG

##### MALDI-TOF MS-based bacterial identification

A thin smear of freshly grown bacterial culture was applied directly onto the spot of MALDI plate at room temperature for one minute (Rahi et al. 2016). Then, 1 µL of the matrix solution, i.e., a saturated solution of *α*-cyano-4-hydrocinnamic acid in 50% acetonitrile HPLC grade and 2.5% trifluoroacetic acid was added to the sample and incubated for 10 min at room temperature. The sample was analyzed using the Autoflex speed system (Bruker Daltonik GmbH, Germany). The mass spectrum of each bacterial isolate was retrieved at 2,000 to 20,000 Da mass range with a laser frequency of 1000 Hz. The external standard calibration mixture, i.e., *Escherichia coli* extracts including RNase A and myoglobin was used for spectral calibration. The MALDI Biotyper software 3.0 (Bruker Daltonik) was used to visualize the mass spectra and identify the isolates. Biotyper score value > 2.0 was considered for species-level identity and > 1.8 was considered for genus-level identity.

##### 16S rRNA gene-based identification of bacterial isolates

Genomic DNA of the bacterial strains was extracted using PureLink^®^ Pro 96 Genomic DNA purification kit (Invitrogen, Inc. USA) from fresh 24-h cultures. PCR amplification of the ~ 1.5 kb 16S rRNA gene was done using universal bacteria specific primers (27F: 5’GAGTTTGATCMTGGCTCAG-3’ and 1492R: 5’-TACGGYTACCTTGTTACGA-3’) (Lane 1991). Each 50 µL PCR reaction contained: 50 ng template DNA, 5 µL 10X reaction buffer, 2.5 µL MgCl_2_ (25 mM), 10 mol of each primers, 1 µL dNTP mix (10 mM) and 1 U Taq polymerase (Life Technologies, USA). The PCR reaction was done with an initial denaturation at 94 °C for 5 min, followed by 30 cycles of denaturation at 94 °C for 60 s, annealing at 58 °C for 45 s, extension at 72 °C for 90 s, and a final elongation step at 72 °C for 10 min. The amplified products were PCR purified using 20% PEG-NaCl (Polyethylene Glycol-NaCl) method. Purified ~ 1.5 kb products were directly sequenced using 536F, 704F and 907R primers by ABI PRISM Big Dye Terminator v3.1 Cycle Sequencing kit on a 3730xl Genetic Analyzer (Applied BioSystems). Bioedit version 7.2.6.1 (Hall 1999) was used for sequence editing and contig formation. Then the sequence similarity search of query sequence data (approximate 1100 bp) was compared with 16S rRNA gene data of public database (NCBI) and Eztaxon by BLAST. For phylogenetic analysis, 16S rRNA gene sequences of different bacterial strains which showed close similarity with query sequences were retrieved from gene bank and Eztaxon database followed by multiple alignments using ClustalW. The resulting alignment was used to construct a phylogenetic tree incorporating the neighbor-joining method and Jukes-Cantor distance matrix by MEGA 7 software package. Bootstrap percentage (1000 bootstrap replicon) was used to check the robustness of the phylogenetic tree formation.

##### Nucleotide sequence accession numbers

Nucleotide gene sequences obtained from this study were deposited in the NCBI GenBank nucleotide sequence database (http://www.ncbi.nlm.nih.gov/genebank/), with the accession numbers MG733189 to MG733194; MG733198 to MG733237; MG733239 to MG733242; MG733244 to MG733255 and MG733269 to MG733274 (Supplementary Table 2).

*Statistical analysis:* All the experimental observations were recorded in triplicate (*n* = 3) and data were represented as mean ± SD. For statistical comparison of the experimental data, analysis of variance (ANOVA) at 5% significance level (*p* < 0.05) was performed. To determine the significant variations, one-way ANOVA followed by Duncan’s multiple range test was performed. Spearman correlation was performed to identify the associations among the variables. Principle Component Analysis (PCA) was also done to identify the structure of the relationship between the speleothem and cave wall deposit samples with respect to their geochemical properties. Another PCA was performed based on bacterial genera distribution within the samples. The interrelationships between constituent bacterial populations and geochemical factors (total organic carbon, calcium, salinity, inorganic carbon) in each sample was determined by Canonical Correspondence Analysis (CCA) using the R environment (Paul et al. 2014)*.*

## Results

### Geochemistry

#### Elemental concentrations in spring waters

Analyses of the drip and spring waters showed various trace and major elements (Supplementary Table 1). The temperature in drip water was 17–19 °C, whereas air temperature inside the cave was 20–23 °C. The pH of the cave samples was neutral (7.1 to 7.7) and the conductivity ranged from 12 to 421 µs with significant variations (*p* < 0.05). The average total carbon content was high (~ 16.44) and significant differences (*p* < 0.05) were noted among the samples. The inorganic carbon content of the water samples varied significantly (*p* < 0.05). It was higher in LBS3 (29.34 ± 0.2 mg L^−1^) and lowest in LBS2 (1.26 ± 0.2 mg L^−1^). The bicarbonate contents in drip and spring water also varied and ranged from 36.6 ± 0.07 to 287.92 ± 0.46 and 129.32 ± 0.13 to 424.56 ± 0.31 mg L^−1^ while the value in the pool water was 500.2 ± 0.1 mg L^−1^. The results showed that Ca^2**+**^ concentrations significantly differ (*p* < 0.05) in drip and spring water samples of different distances and zones along the length of the cave and ranged from 4.8 ± 0.2 to 30.4 ± 0.16 mg L^−1^ and 16.0 ± 0.68 to 43.2 ± 0.14 mg L^−1^, respectively. Significant (*p* < 0.05) disparities were observed in sulphate (0.01 ± 0.01 to 21.95 ± 0.86 mg L^−1^) and nitrate (0.06 ± 0.02 to 4.6 ± 0.2 mg L^−1^) contents. Significant variations (*p* < 0.05) were observed in magnesium (7.77 ± 0.07 to 98.1 ± 0.02 mg L^−1^), sodium (1.3 ± 0.1 to 6.2 ± 0 mg L^−1^), chloride (7.1 ± 0.05 to 19.02 ± 0.01 mg L^−1^), total hardness (44 ± 0.29 to 452 ± 0.1 mg L^−1^), total dissolved solids (18.9 ± 0.02 to 299 ± 0.12 mg L^−1^) and salinity (11.3 ± 0.1 to 189 ± 0.69 mg L^−1^) contents in the drip, pool and spring waters (Supplementary Table 1).

### Microbiology

#### Isolation and identification of microorganisms

A total of 826 bacterial strains were isolated from all the cave samples on different media. Except for KSSTc6, bacterial colonies were observed in most of the cave samples cultured on dilute Nutrient Agar (NA), M9 and R2A medium. KSSTc1, LBSTc1, LBSTc2. LBWDc1 showed no growth on dilute NA and M9 media (Table 1). Speleothems from all three caves showed high microbial enumerations on dilute nutrient agar (5.3 × 10^3^ to 8.8 × 10^5^) followed by M9 minimal medium (4 × 10^4^ to 1.7 × 10^5^) and R2A medium (1.0 × 10^4^ to 5.78 × 10^5^). From the above media, 295 isolates were identified using MALDI-TOF (227 isolates) and 16S rRNA gene (68 isolates)-based sequencing.

**Fig. 1:**
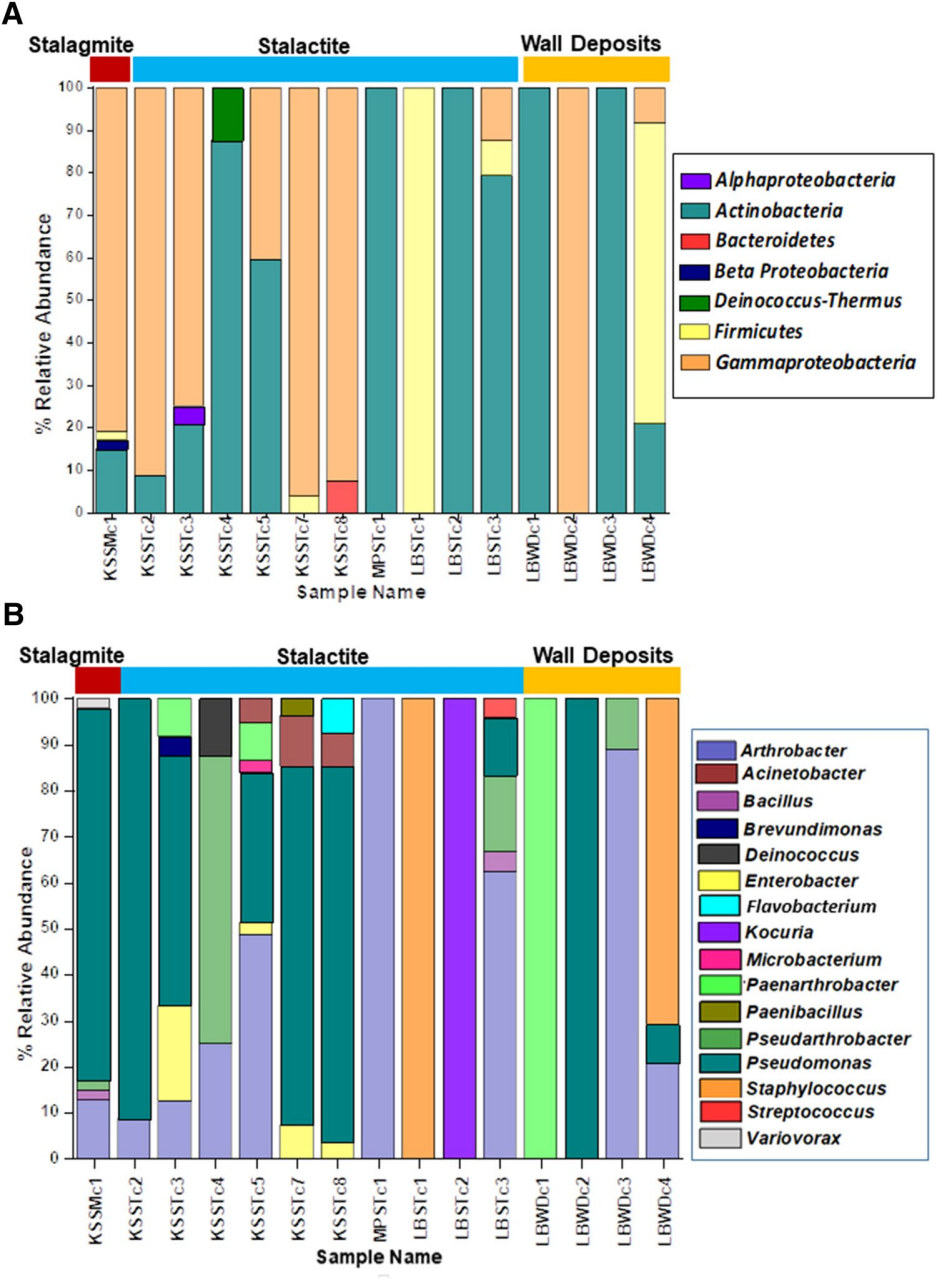
Distribution of major phylogenetic groups of bacteria. **A** Abundance of bacterial groups is plotted with respect to their affiliation at phylum level. **B** Abundance of bacterial groups is plotted with respect to their affiliation at genus level

Taxonomic analysis showed that the bacterial isolates belonged to 16 genera under 5 phyla. Taxonomic analysis based on relative abundance revealed that Proteobacteria (61%) was the dominant bacterial group followed by Actinobacteria (30%), Firmicutes (7.45%), Bacteroidetes (0.67%) and Deinococcus-Thermus (0.33%; Fig. 1a; Supplementary Table 3).

Proteobacteria and Actinobacteria were the most abundant bacterial community (91%, Fig. 1a). The bacterial phyla Proteobacteria (1.1–21.5%) was ubiquitously present in all the speleothems samples except KSSTc4, MPSTc1, LBSTc1, LBSTc2, LBWDc1, and LBWDc3 from Krem Soitan and Krem Lawbah. Within the Proteobacteria, Alpha-, Beta- and Gamma-proteobacteria were found in the samples. Gammaproteobacteria were the most abundant class (8–96%) detected in most of the samples. Alphaproteobacteria (4.1%) and Betaproteobacteria (2.1%) were found in samples KSSTc3 and KSSMc1, respectively. Actinobacteria (1.1–24.7%) was also observed in most of the samples except KSSTc1, KSSTc7, KSSTc8 from Krem Soitan and LBSTc1, LBWDc2 from Krem Lawbah. Bacterial members Deinococcus-Thermus and Bacteroidetes were present as a minor group and were exclusive to the stalactite samples of Krem Soitan. Other significant phyla which constituted > 5% of the sequences included the Firmicutes (7.45%). The phylum Firmicutes was present in the stalactites and stalagmites of Krem Soitan and Krem Lawbah (Supplementary Table 3).

The bacterial genera *Acinetobacter, Arthrobacter, Bacillus, Brevundimonas, Deinococcus, Enterobacter, Flavobacterium, Kocuria, Microbacterium, Paenarthrobacter, Paenibacillus, Pseudarthrobacter, Pseudomonas, Staphylococcus, Streptococcus* and *Variovorax* were also identified in the cave samples (Fig. 1b; Supplementary Table 4). A total of 71 bacterial species were identified from all the cave deposits. *Pseudomonas* (55%) and *Arthrobacter* (23%) were the most abundant genera (9 out of 15 samples) in the study (Supplementary Table 4). The genus *Arthrobacter* was detected in all three cave samples whereas *Pseudomonas* was noted in Krem Soitan and Krem Lawbah only. Among the *Pseudomonas* and *Arthrobacter*, the following strains were identified. They include: *A. oxydans, A. oryzae, Pseudarthrobacter oxydans, P. koreensis, P. chlororaphis, P. granadensis, P. alkylphenolica,* and *Paenarthrobacter nicotinovorans* (Fig. 1b; Supplementary Table 5).

#### Diversity in Krem Soitan

Maximum numbers of isolates were recovered from stalagmite KSSMc1 (47 strains) followed by stalactite KSSTc5 (37 strains) (Supplementary Table 4). Among the Proteobacteria, members belonging to Alphaproteobacteria, Betaproteobacteria and Gammaproteobacteria were observed. The members noted were *Enterobacter, Pseudomonas, Brevundimonas* and *Variovorax* genera. Among the *Enterobacter* genera, *E. asburiae, E. cloacae, E. lugwigii* and *E. tabaci* were identified. Twenty four bacterial strains of *Pseudomonas chlororaphis* (51%) was observed in the stalagmite (KSSMc1). Among the family Comamonadaceae, *Variovorax paradoxus* was exclusive to the stalagmite, KSSMc1. One strain of *Brevundimonas vesicularis* was also identified (KSSTc3) as a relatively minor population (< 1%). Among the *Actinobacteria*, strains belonging to *Arthrobacter* sp*., Pseudoarthrobacter* sp., *Paenarthrobacter* sp.*, Kocuria* sp., *Microbacterium* sp*.,* were observed. Eighteen strains belonged to genus *Arthrobacter* in KSSTc5 (48%; Fig. 1b). *A. aurescens, A. ginsengisoli, A. histidinolovorans, A. ilicis, A. methylotrophus, A. nicotinovorans, A. oxydans, A. pascens, A. polychromogenes, A. sulfonivorans* were the main strains identified. *Pseudarthrobacter oxydans* was also observed in the stalactite and stalagmite. Five strains of *Paenarthrobacter nicotinovorans* were also identified in the stalactites. It was noted that < 1% of the bacterial genera belonged to *Deinococcus, Microbacterium* and *Paenibacillus*. *Kocuria* belonging to Micrococcaceae, were also detected as a minor population (< 1%). Within the Acinetobacter genus, four strains of *A. johnsonii,* three strains of *A. woffii* were identified in stalactite samples. Among the Bacteroidetes and Firmicutes, *Flavobacterium* was noted in only one stalactite sample (KSSTc8) and *Bacillus* genera was observed in two samples, (KSSMc1 and KSSTc7) from Krem Soitan. One strain of *Deinococcus ficus* was identified from the stalactite (KSSTc4).

#### Diversity in Krem Mawpun

Among the Actinobacteria, 11 strains belonging to *Arthrobacter* sp. including 7 strains of *A. oxydans* and 2 each of *A. polychromogenes* and *A. sulfonivorans* were identified from the stalactite (MPSTc1; Supplementary Table 5).

#### Diversity in Krem Lawbah

Thirty one strains were identified from the wall deposit LBWDc2 and 24 strains each from LBWDc4 and LBSTc3. Among the Proteobacteria, all the 31 strains identified from the wall deposit LBWDc2, belonged to the genus *Pseudomonas*. The strains *P. granadensis, P. koreensis, P. chlororaphis, P. jessenii,* were observed from wall deposits LBWDc4 and LBWDc2. Among the genus *Arthrobacter*, 19 strains were observed (79%). *A. oxydans* (5 strains)*, A. pascens* (2 strains), *A. polychromogenes* (6 strains), *Pseudarthrobacter polychromogenes* (4 strains); *A. ginsengisoli, A. sulfonivorans* (1 strain each) were identified from stalagmite (LBSTc3) (Supplementary Table 5). Minor percentages of the members belonging to the phylum Firmicutes was present in samples LBSTc1, LBSTc3, LBWDc4. Among the Firmicutes*, Bacillus, Staphylococcus* and *Streptococcus* genera were observed. One strain of *Staphylococcus hominis* was identified from stalactite LBSTc1 (< 1%) and 17 strains belonged to *Staphylococcus warneri* from wall deposits LBWDc4. *Streptococcus gallolyticus* was noted in LBSTc3 (< 1%). *Bacillus safensis* was present in one sample (LBSTc3) (Supplementary Table 5).

**Fig. 2:**
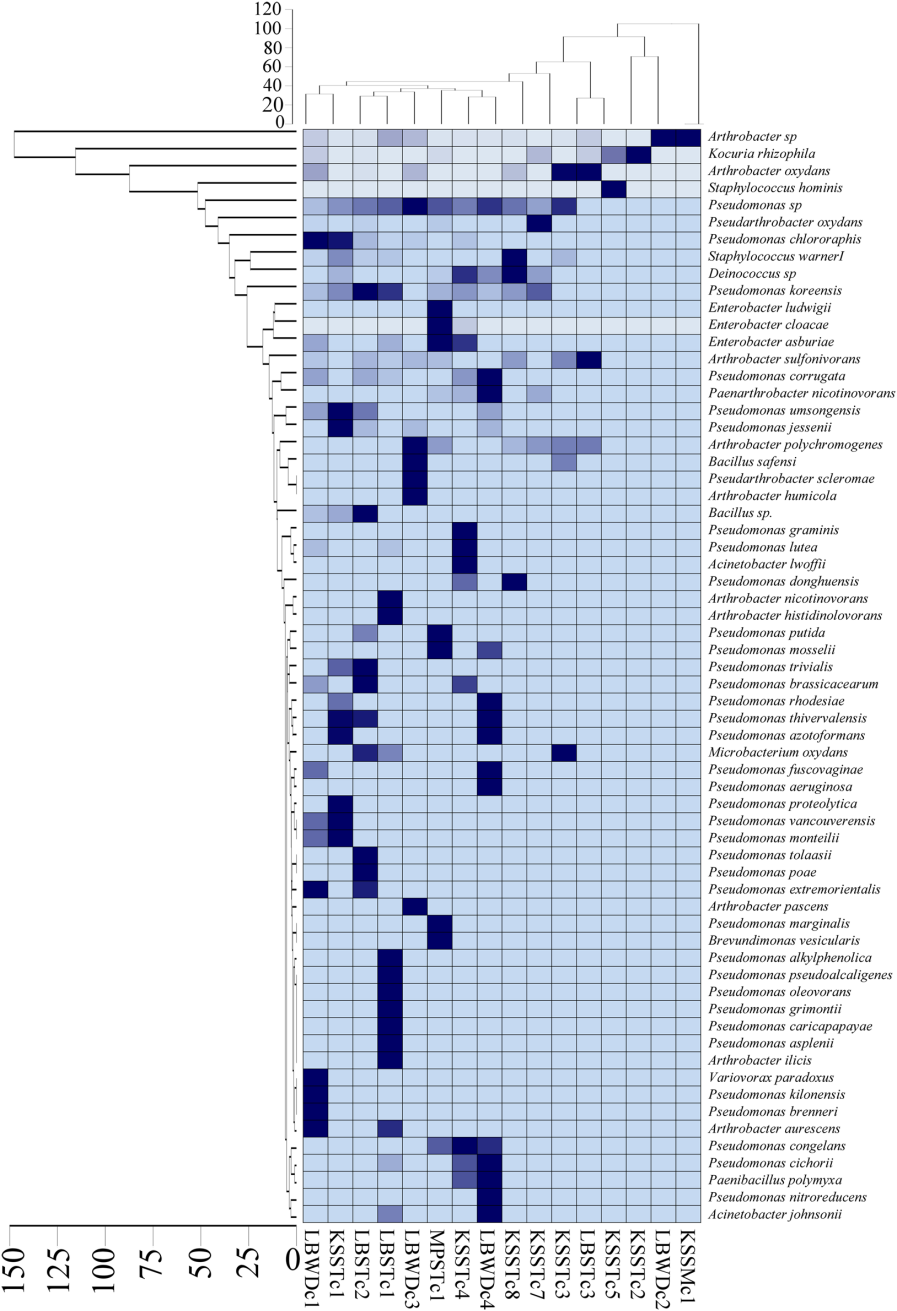
Heat map shows species level distribution of major bacterial group of the studied cave samples

To identify the relationship among the samples based on species level distribution, the UPGMA (unweighted pair group method with arithmetic mean) cluster analysis was performed. Analysis revealed that most of the samples from the same cave formed a separate cluster (Fig. 2). Most of the Krem Soitan samples clustered together except KSSMc1, KSSTc1 and KSSTc4. Similarly, samples from Krem Lawbah grouped together at 40% similarity level except sample LBWDc1 (Fig. 2).

**Fig. 3:**
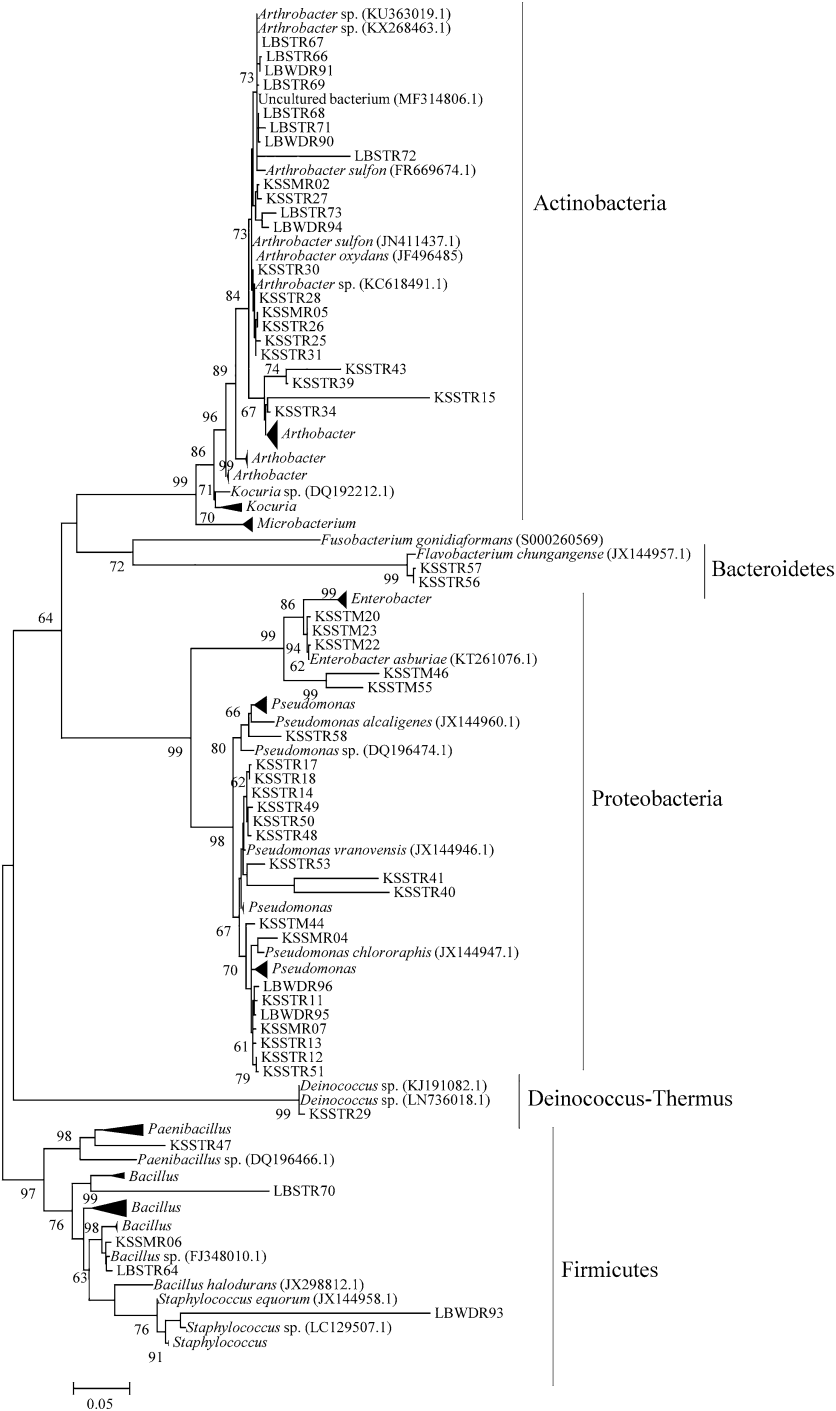
Maximum likelihood phylogenetic tree of isolated bacterial strains of the present study. The tree is constructed based on 16S rRNA gene sequences using Jukes-Cantor distances. 1000 bootstraps analyses are conducted and more than 50% are denoted in nodes

Phylogenetic analysis as ascertained by the neighbour-joining tree where the close lineage of bacteria strains was retrieved by doing BLAST search in Eztaxon database (Fig. 3). It was found that bacterial strain KSSMR06 and LBSTR64 showed close lineage with type strain *Bacillus altitudinis* and *Bacillus safensis*, respectively (Supplementary Fig. 3). Bacterial strains KSSTR28, KSSTR31, KSSTR25, KSSTR26, LBSTR73 showed high sequence identity among themselves and branched together with type strain *Arthrobacter ginsengisoli*. Similarly, bacterial strains LBSTR66, LBWDR91, LBWDR90, and LBSTR67 form a separate clade with type strain *Pseudarthrobacter oxydans* (Supplementary Fig. 4). Most of the bacterial strains belonging to Proteobacteria showed close lineage with type strains *Pseudomonas hutmensis* and *Pseudomonas kribbensis* (Supplementary Fig. 5). In addition bacterial strain KSSTM20, KSSTM23 formed clade with *Enterobacter sichuanesis* belonging to Gammaproteobacteria and KSSTR29 formed clade with type strain *Deinococcus ficus* (Supplementary Fig. 5).

Phylogenetic analysis also indicated other bacterial strains such as LBSTR70, KSSTR47, KSSTR43, KSSMR04, KSSTM46, and KSSTM44. These strains form a separate clade in the phylogenetic tree. It was found from BLASTN search in NCBI database (Supplementary Table 6) that they showed 92–98% similarity with their respective closely related organisms. This may indicate that these are novel strains of the closely related organisms. Hence, they may form separate clades in the phylogenetic tree (Fig. 3 and Supplementary Fig. 3–5). In addition to this, some of the strains (LBWDR74, LBWDR90, LBWDR94, LBWDR93) isolated from cave wall deposits showed 83–99% similarity with their respective closely related organisms isolated from other caves (Supplementary Table 7).

### Association among the taxa and geochemical parameters

**Fig. 4:**
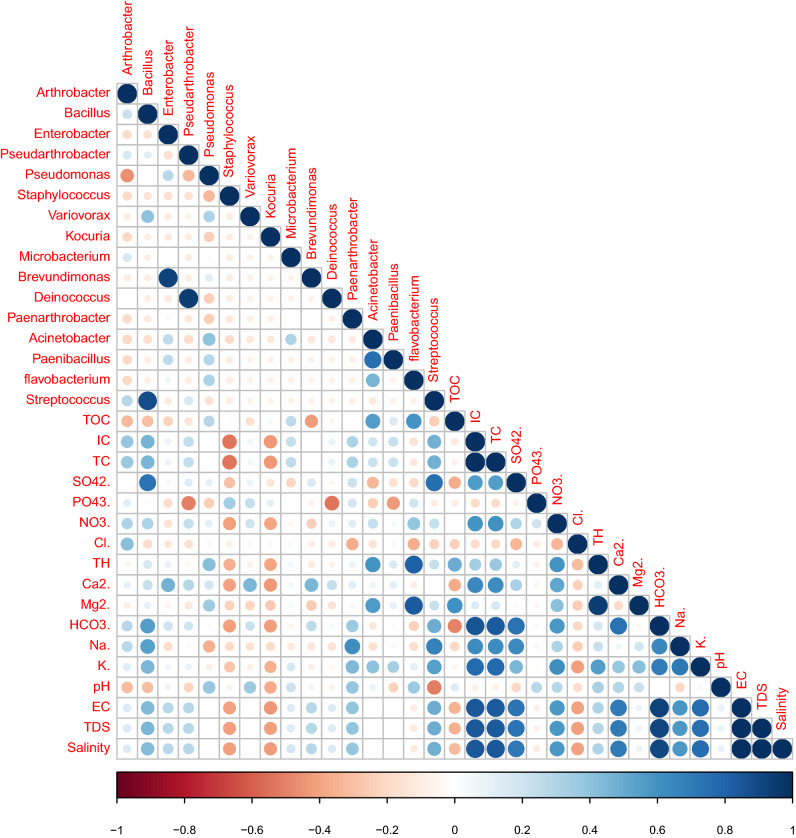
Correlation analysis between bacterial members and physicochemical properties in water samples. Strong correlations are indicated by large circles, whereas weak correlations are indicated by small circles. The color of the scale bar denotes the nature of the correlation, with + 1 indicating a perfect positive correlation (dark blue) and − 1 indicating a perfect negative correlation (dark red)

Correlation analysis showed that *Enterobacter, Variovorax, Acinetobacter, Paenibacillus, Flavobacterium* genera had a positive association with *Pseudomonas* whereas *Arthrobacter, Staphylococcus, Deinococcus* and *Paenarthrobacter* genera showed a negative association with *Pseudomonas* (Fig. 4). Association analysis among the bacterial genera and geochemical factors indicated that *Bacillus, Enterobacter, Pseudarthrobacter, Brevundimonas* and *Streptococcus* showed a positive association with electrical conductivity, total dissolved solids, and salinity of the samples whereas *Staphylococcus* and *Kocuria* showed a negative association with these geochemical factors. Most of the predominant bacterial groups, i.e., *Arthrobacter, Bacillus, Pseudoarthobacter, Microbacterium, Paenarthobacter, Acinetobacter, Paenibacillus* and *Streptococcus* showed moderate to strong positive association with inorganic carbon and total carbon. In contrast, *Staphylococcus* and *Kocuria* showed a negative association. *Arthrobacter, Bacillus, Paenarthrobacter* and *Streptococcus* showed a positive association with HCO_3_, Na and K whereas *Staphylococcus* and *Kocuria* showed a negative correlation with these factors. It was also noted that *Staphylococcus* and *Kocuria* showed a negative association with several parameters namely nitrate, calcium, bicarbonates, electrical conductivity, total dissolved solids, salinity, inorganic carbon, total carbon, total hardness, and magnesium of the samples. Interestingly, it was found that bacterial genera *Pseudomonas* showed the opposite trend with bacterial genera *Arthrobacter* in case of most of the samples. The pattern of this organism is self-explanatory with the geochemical approach. Implementing correlation analysis within the geochemical factor and microbial genera, showed that *Arthrobacter* has a positive association with geochemical factors TC, IC, NO_3_, Cl^**−**^, HCO_3_, Na and a negative correlation with TOC and pH. *Pseudomonas* showed the opposite trend with these geochemical factors, i.e., positive association with TOC and pH and a negative correlation with TC, IC, NO_3_, Cl^**−**^, HCO_3_, Na (Fig. 4). This indicates that geochemical factors plays a significant role in shaping microbial patterns in this ecosystem.

**Fig. 5:**
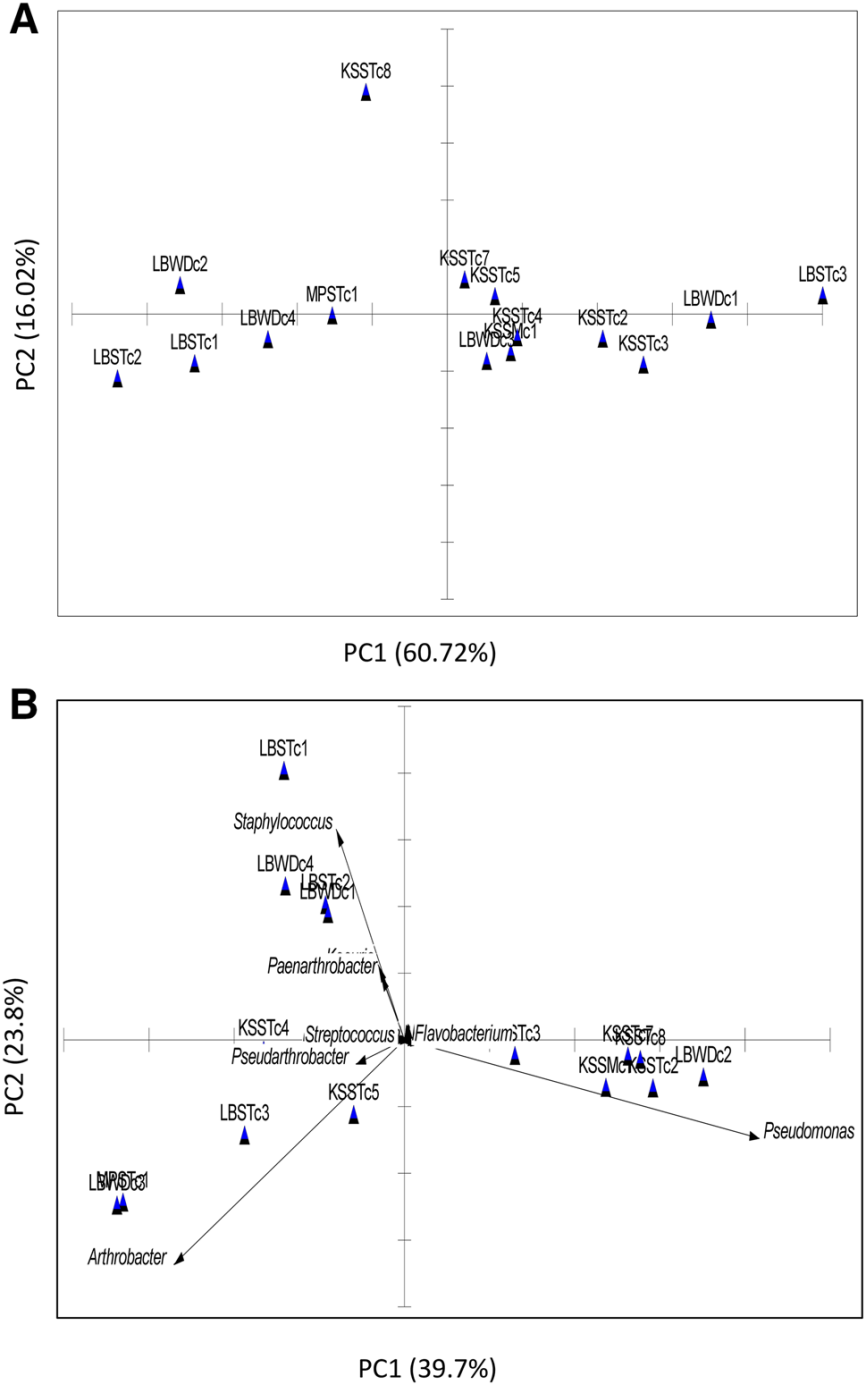
PCA and biplot PCA based ordination plots on water samples microbiological and physicochemical properties. **A** PCA of the water samples based on geochemical parameter; **B** bi-plot PCA of the water samples based on genus level distribution

The interrelation among the samples with respect to their geochemical properties and bacterial community composition were analysed by statistical analyses. PCA performed on selected water geochemistry parameters revealed that samples LBSTc1, LBSTc2 and LBWDc4 formed a cluster, whereas samples KSSMc1, KSSTc2, KSSTc3, KSSTc4, LBWDc1 and LBWDc3 were related to these samples (Fig. 5a). Based on abundance of bacterial genera, a biplot PCA was performed. Axes 1 and 2 of the resulting bi-plot gave 39.7% and 23.8% of the total variability. It was also observed that sample LBSTc1, KSSTc4, LBSTc2, LBWDc4 and LBWDc1 grouped together as *Staphylococcus* and *Paenarthrobacter* whereas KSSMc1, KSSTc7, KSSTc8, KSSTc2 and LBWDc2 formed another separate cluster including *Pseudomonas, Flavobacterium* (Fig. 5b).

## Discussion

Caves are unique ecological niches for conducting geomicrobiological investigations. Diverse microbial communities exist in caves, which are important in various microbe-mineral processes (Tomczyk-Żak and Zielenkiewicz 2016). The cave walls in the different zones are often characterized by distinct colours, biofilms, and odours characteristic of certain microbes (Lavoie et al. 2017). Bacterial community structure and their functional activities can specifically contribute to cave ecology. This report is the first study on the culturable diversity from caves in Khasi hills, Meghalaya using MALDI-TOF spectrometry followed by 16S rRNA gene sequencing. The caves analysed for the study have speleothems with remarkable states of preservation. Our earlier study in the same caves evidenced diverse microbial fossil forms and structures that included coccoid-like shells, reticulate filaments, flat and beaded forms of filaments, and conidiophore-like structures (Mudgil et al. 2018). Although geomicrobiological studies have been reported from some caves in Meghalaya (namely Krem Mawsmai, Krem Phyllut (Baskar et al. 2009); Krem Mawluh (Baskar et al. 2011; Banerjee and Joshi 2016), a detailed insight on the culturable microbial community composition are provided in the present study.

### Geochemical parameters and microbial community distribution in caves

Physicochemical analyses of water samples based on pH, Ca^2+^, salinity, total organic carbon, Na^+^, Cl^−^, and SO_4_^2−^ and abundance of HCO_3_^−^ showed that these caves have comparable chemical characteristics of other cave ecosystems (Raeisi et al. 2013). Geochemical processes like evaporation, root-zone CO_2_ enrichment, bedrock dissolution, CO_2_ degassing and speleothems precipitation might play a significant role in the distribution of these major ions. Total carbon content of the speleothems samples is low (< 2 mgL^−1^), indicating that the analysed caves are nutrient-limited environments. The source of the carbon could be from percolating water through rocks from the soil above the caves (Simon et al. 2007). Nitrate (≤ 3.52 mg L^−1^) was also detected in the present study, like that reported by Menció et al. 2016. Thus, caves serve as a source of nutrients and energy for a variety of microbial communities. Increased nutrient availability may also result in the Proteobacterial dominance (Tomczyk-Żak and Zielenkiewicz, 2016).

### Diversity of microbial communities in caves

Very few studies have examined the culturable bacterial populations in cave environments. Most of the key bacterial genera identified in this study are also reported in other limestone caves (Rusznyák et al. 2012; Ortiz et al. 2013). In the present study, cave samples showed higher abundance of Proteobacteria followed by Actinobacteria, Firmicutes, Bacteroidetes and Deinococcus-Thermus. They are well reported and known for the metabolic potentiality to maintain nutrient cycles in nutrient limited cave environment (Balkwill et al. 1997). The predominance of Proteobacterial representatives and their high metabolic growth rates may be due to the soil carbon inputs in the caves using these sources as substrates. Some microbial strains can mobilize inorganic phosphates, hydrolyse proteins and lipids produced by other microbes thereby allowing recycling of resource materials (Barton and Jurado 2007). Saprophytic microorganisms such as Firmicutes and Actinobacteria are important in soil decomposition and formation (Anandan et al. 2016). The variation of these phyla in the different samples analysed may be due to changes in the ratio of obligate aerobes and facultative anaerobes in relation to the carbon source (Itcus et al. 2018). Further the different organic carbon inputs could be associated to the relative abundances of the observed bacterial genera. These can be some reasons for the specific clusters of microbes formed with geochemical parameters and associations between bacterial genera.

#### Members belonging to Proteobacteria

Proteobacteria is the most abundant phylum and represented 61% of the bacterial populations. Within the Proteobacteria, Alpha-, Beta- and Gamma-proteobacteria were found in the samples. Gammaproteobacteria were the most abundant class detected in most of the samples followed by Alphaproteobacteria and Betaproteobacteria. This indicates that Gammaproteobacteria might be play a significant role in most of the caves of the present study. Our analyses indicate that the most abundant and detectable populations (*Pseudomonas, Enterobacter, Variovorax, Brevundimonas*) are distinct from the populations identified in other caves (Wu et al. 2015). Most of the caves world-wide are dominated by Proteobacterial populations and are well known for their chemoorganotropic/ chemolithotrophic metabolisms (Rusznyák et al. 2012). In our study, the Proteobacterial members, *Pseudomonas* sp.*, Enterobacter* sp. are closely associated with the strains identified from caves in India, Slovenia, Kartchner caverns Arizona, USA. *Variovorax paradoxus* belongs to the Betaproteobacteria, known for sulphur transformations (Northup et al. 2003), and are exclusive to the stalagmite sample at Krem Soitan. Bacterial genera *Brevundimonas* (Alphaproteobacteria) identified in the study have high survival rates in extreme environments. Therefore, the prevalence of Proteobacteria in the present study is consistent with studies in other karst cave ecosystems, such as in southwest China (Zhu et al. 2019) and from the Italian Pertosa-Auletta Cave (Adesso et al. 2020), evidences their importance in different biogeochemical cycles in the ecosystem.

#### Members belonging to Actinobacteria

Actinobacteria was the second most predominant bacterial group in our cave samples. These genera are also reported in caves from Slovenia, Germany (Rusznyák et al. 2012), caves in Slovenia (Pašić et al. 2010). Actinobacteria are known for carbonate biomineralization in caves and other ecosystems (Baskar et al. 2014; Mudgil et al. 2018. *Arthrobacter methylotrophus* identified in our study is a facultative methylotroph and has been reported in the ferromanganese deposits in caves situated in the Upper Tennessee River Basin (Carmichael et al. 2013).

#### Members belonging to Firmicutes

Bacterial members belonging to Firmicutes were found in most of the samples*.* The predominance of *Bacillus, Paenibacillus* and *Staphylococcus* species have been reported in the phototrophic biofilms in the Cave of Bats (Urzì et al. 2010). *Bacillus* sp. easily resist stress conditions and can survive in extreme conditions due to endospore formations (Filippidou et al. 2016). Several studies have reported the prevalence of Firmicutes in caves and their role in maintaining homeostatic conditions in caves (Herrenberg Cave, Rusznyak et al. 2012 and Weebubbie, Tetu et al. 2013).

#### Members belonging to Bacteroidetes

Among the Bacteroidetes, *Flavobacterium tructae* and *F. hercynium* which were identified in this study have Mn mineral precipitating abilities in vitro (Carmichael et al. 2013). These organisms are identified as the largest group in biofilms on ferromanganese deposits in the Carter Saltpeter Cave (Carmichael et al. 2013). Bacteroidetes have been noted as the second largest group of microbes in Altamira Cave, represented dominantly by *Flavobacterium* (Portillo et al. 2009). *Deinococcus ficus,* identified in this study are members of Deinococcus-Thermus, and are extremophiles, chemoorganoheterotrophs, and ionizing-radiation resistant bacteria (Lai et al. 2006).

Read et al. (2021) in their study reported on the bacterial diversity of 17 pools in 3 New Mexican arid land caves. Even while the pools had the same basic water chemistry, no two pools had the same communities, even at the phylum level (Read et al. 2021). They further claim that each pool is a distinct, isolated ecosystem, with variances owing to the pool's isolation rather than differences in water chemistry. These findings also suggest that future cave research should not group samples to appropriately estimate the diversity present in cave ecosystems (Read et al. 2021).

The identification of diverse microbial communities in this study suggest that in subsurface environments such as caves, various groups of microbes work together creating conducive environments for microbe-mineral interaction.

## Conclusions

The present study evidences the presence of several strains novel to caves which expand our knowledge regarding microbial diversity in these geomicrobiologically unexplored habitats. A dominance of Proteobacteria was observed and is in accordance with other cave studies. Further, the correlation between geochemical parameters and microbial community composition indicates that geochemical parameters strongly influence the distribution of microbial communities. Future studies should focus on microbial roles in biogeochemical cycles and cave population dynamics. Such studies will be an all-inclusive approach for the taxonomical and functional profiling. These can be helpful in bioprospecting potential molecules such as enzymes/and antibiotics for industrial and pharmaceutical applications.

## Supplementary Information

Below is the link to the electronic supplementary material.

Supplementary file1 Supplementary Fig. 1 Location map of Meghalaya showing study area (a) Geological map of Meghalaya (modified and adapted from National Bureau of soil survey and land use planning, 1996). (b) Location map of Krem Lawbah, Krem Mawpun, Krem Soitan and Mawlyngbna; map of Meghalaya in Insat showing 8 Blocks (1–8) of East Khasi hills. (PNG 1014 KB)

Supplementary file2 Supplementary Fig. 2 Speleothems sampled for the study from caves in Khasi hills, Meghalaya (a–c) Krem Mawpun; (a) Cave entrance (b, c) soda straws and stalactites sampled (MPST1); (d–g) Krem Soitan (d) Entrance of Krem Soitan cave (e) dendrite-shaped stalactite (KSST3); (f) Moonmilk deposit (KSST7); (g) Cave popcorns (KSSM1); (h–j) Krem Lawbah; (h) stalactite (LBWD1); (i) Flowstone on cave wall (LBWD4) (j) column (LBST3). (PNG 3900 KB)

Supplementary file3 Supplementary Fig. 3 Neighbor-joining phylogenetic tree of Bacteroidetes, Deinococcus and Firmicutes. 16S rRNA gene-based tree reflecting the phylogenetic relationships of strains identified and a selection of reference sequences. The phylogenetic tree was constructed using p-distance matrix of neighbour-joining algorithm with 1000 bootstrap values and visualized by tree view. (PNG 24 KB)

Supplementary file4 Supplementary Fig. 4 Neighbor-joining phylogenetic tree of Actinobacteria.16S rRNA gene-based tree reflecting the phylogenetic relationships of strains identified and a selection of reference sequences. The phylogenetic tree was constructed using p-distance matrix of neighbour-joining algorithm with 1000 bootstrap values and visualized by tree view. (PNG 27 KB)

Supplementary file5 Supplementary Fig. 5 Neighbor-joining phylogenetic tree of Proteobacteria.16S rRNA gene-based tree reflecting the phylogenetic relationships of strains identified and a selection of reference sequences. The phylogenetic tree was constructed using p-distance matrix of neighbour-joining algorithm with 1000 bootstrap values and visualized by tree view. (PNG 24 KB)

Supplementary file6 (DOCX 22 KB)

Supplementary file7 (DOCX 17 KB)

Supplementary file8 (DOCX 14 KB)

Supplementary file9 (DOCX 14 KB)

Supplementary file10 (DOCX 24 KB)

Supplementary file11 (DOCX 13 KB)

Supplementary file12 (DOCX 16 KB)
