# Supplementary figures and images for: Cultivable microbial diversity in speleothems using MALDI-TOF spectrometry and DNA sequencing from Krem Soitan, Krem Lawbah, Krem Mawpun, Khasi Hills, Meghalaya, India
Source: Arch Microbiol. 2022 Jul 17;204(8):495. doi: 10.1007/s00203-022-02916-8 (PMC9288962; doi:10.1007/s00203-022-02916-8)

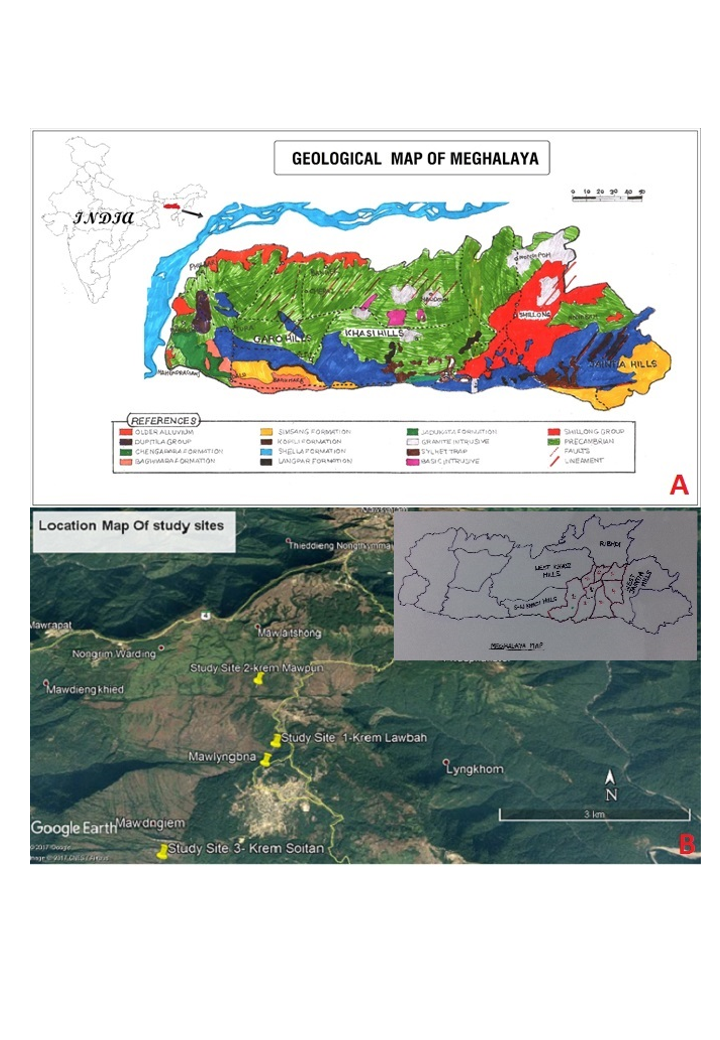

Supplement: Supplementary file 1 — Supplementary file1 Supplementary Fig. 1 Location map of Meghalaya showing study area (a) Geological map of Meghalaya (modified and adapted from National Bureau of soil survey and land use planning, 1996). (b) Location map of Krem Lawbah, Krem Mawpun, Krem Soitan and Mawlyngbna; map of Meghalaya in Insat showing 8 Blocks (1–8) of East Khasi hills. (PNG 1014 KB) [file 203_2022_2916_MOESM1_ESM.png]

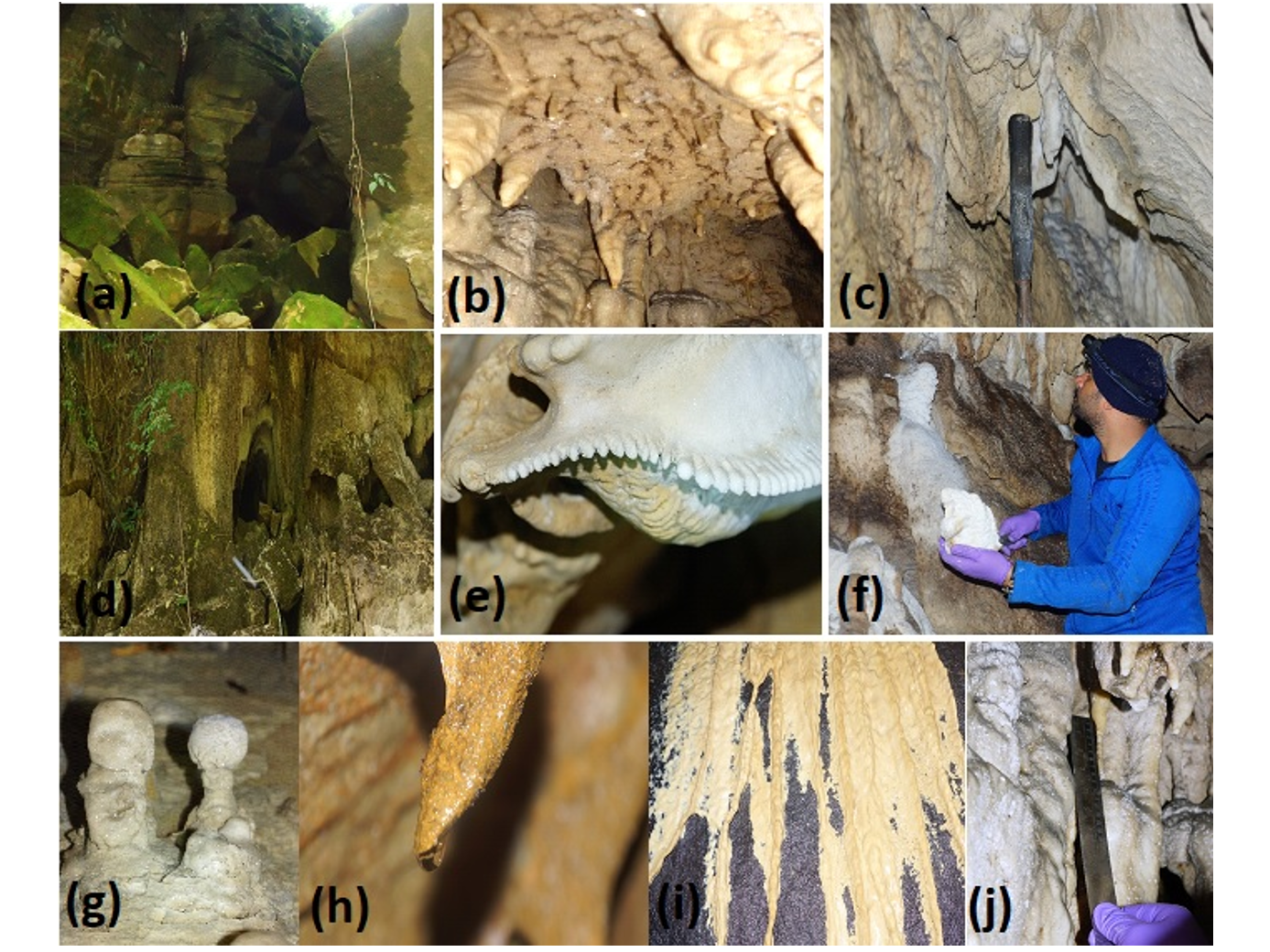

Supplement: Supplementary file 2 — Supplementary file2 Supplementary Fig. 2 Speleothems sampled for the study from caves in Khasi hills, Meghalaya (a–c) Krem Mawpun; (a) Cave entrance (b, c) soda straws and stalactites sampled (MPST1); (d–g) Krem Soitan (d) Entrance of Krem Soitan cave (e) dendrite-shaped stalactite (KSST3); (f) Moonmilk deposit (KSST7); (g) Cave popcorns (KSSM1); (h–j) Krem Lawbah; (h) stalactite (LBWD1); (i) Flowstone on cave wall (LBWD4) (j) column (LBST3). (PNG 3900 KB) [file 203_2022_2916_MOESM2_ESM.png]

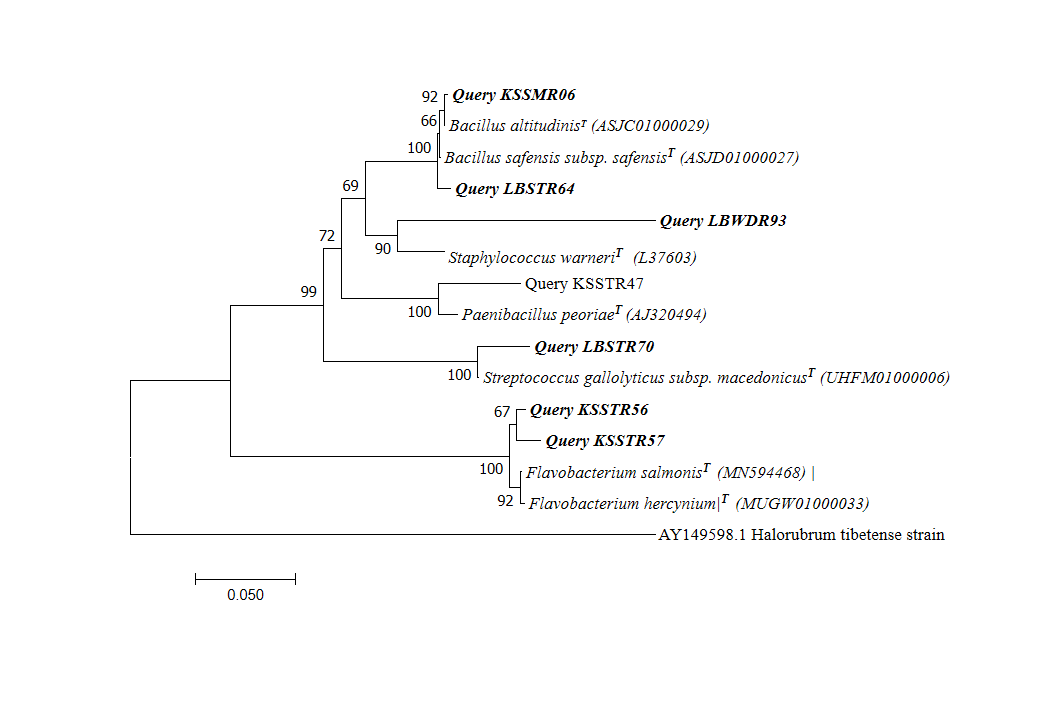

Supplement: Supplementary file 3 — Supplementary file3 Supplementary Fig. 3 Neighbor-joining phylogenetic tree of Bacteroidetes, Deinococcus and Firmicutes. 16S rRNA gene-based tree reflecting the phylogenetic relationships of strains identified and a selection of reference sequences. The phylogenetic tree was constructed using p-distance matrix of neighbour-joining algorithm with 1000 bootstrap values and visualized by tree view. (PNG 24 KB) [file 203_2022_2916_MOESM3_ESM.png]

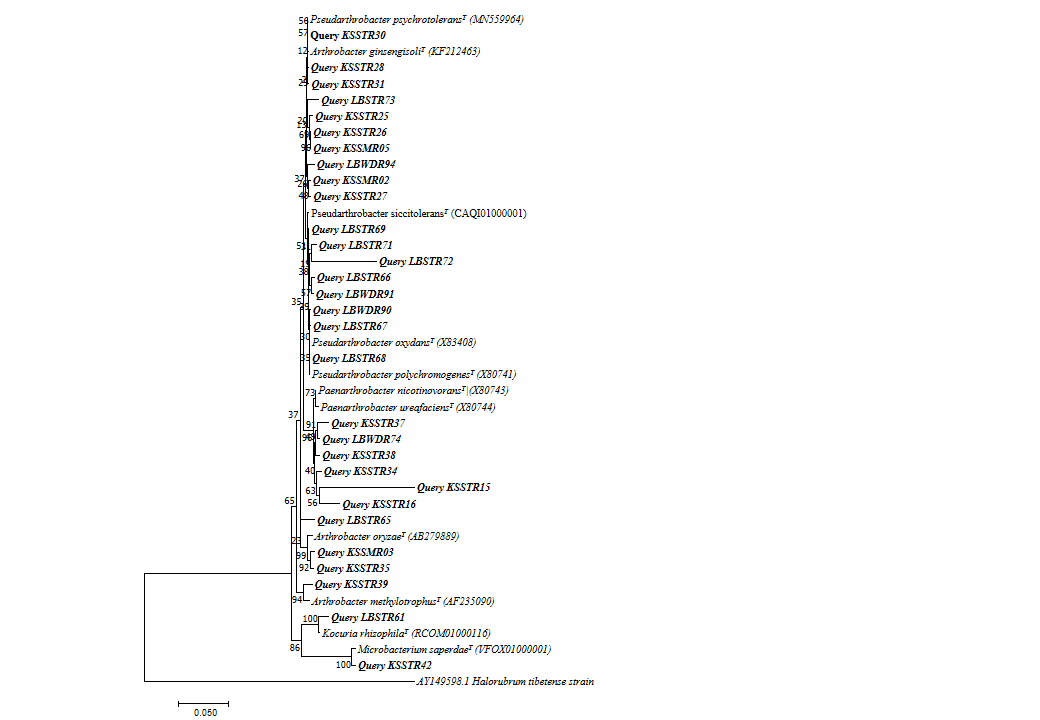

Supplement: Supplementary file 4 — Supplementary file4 Supplementary Fig. 4 Neighbor-joining phylogenetic tree of Actinobacteria.16S rRNA gene-based tree reflecting the phylogenetic relationships of strains identified and a selection of reference sequences. The phylogenetic tree was constructed using p-distance matrix of neighbour-joining algorithm with 1000 bootstrap values and visualized by tree view. (PNG 27 KB) [file 203_2022_2916_MOESM4_ESM.png]

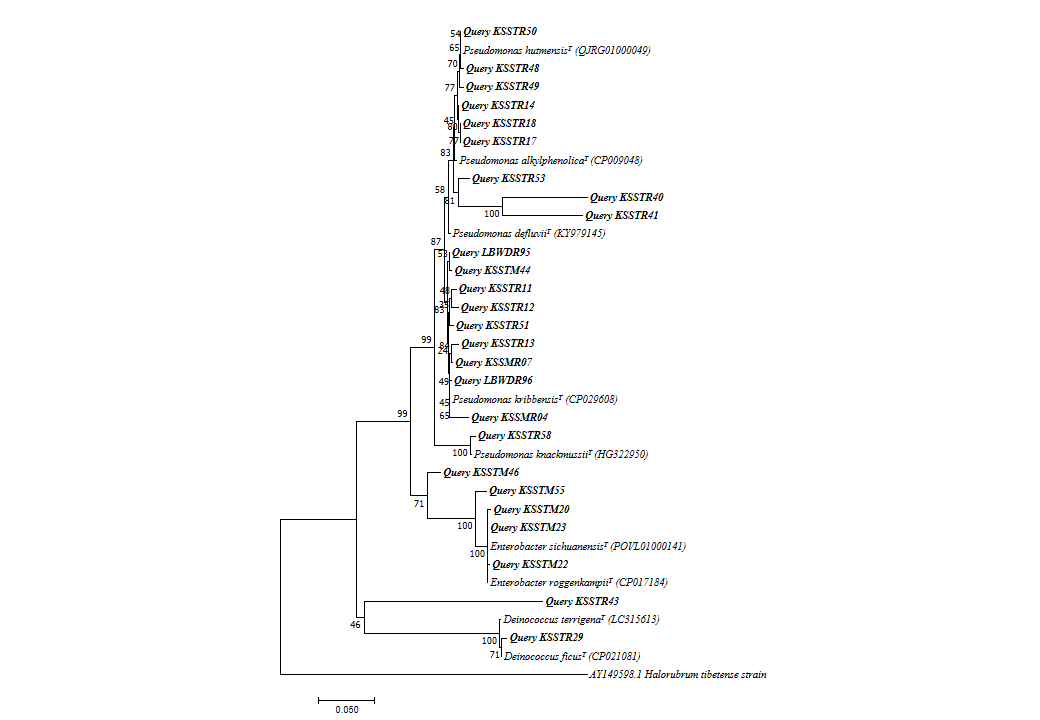

Supplement: Supplementary file 5 — Supplementary file5 Supplementary Fig. 5 Neighbor-joining phylogenetic tree of Proteobacteria.16S rRNA gene-based tree reflecting the phylogenetic relationships of strains identified and a selection of reference sequences. The phylogenetic tree was constructed using p-distance matrix of neighbour-joining algorithm with 1000 bootstrap values and visualized by tree view. (PNG 24 KB) [file 203_2022_2916_MOESM5_ESM.png]
