# Supplementary material for: Cultivable microbial diversity in speleothems using MALDI-TOF spectrometry and DNA sequencing from Krem Soitan, Krem Lawbah, Krem Mawpun, Khasi Hills, Meghalaya, India
Source: Arch Microbiol. 2022 Jul 17;204(8):495. doi: 10.1007/s00203-022-02916-8 (PMC9288962; doi:10.1007/s00203-022-02916-8)
Supplement: Supplementary file 6 — Supplementary file6 (DOCX 22 KB) [file 203_2022_2916_MOESM6_ESM.docx]

| **Sample ID** | **Type of water sample** | **Approx. distance from cave entrance (m)** | **Zone of collection** | | **Sulphate (mg/l)** | | **Phosphate (mg/l)** | | **Calcium (mg/l)** | | **Chloride (mg/l)** | | **EC (µs/cm)** | | **Bicarbonate (mg/l)** | | **Inorganic carbon (mg/l)** | |
| --- | --- | --- | --- | --- | --- | --- | --- | --- | --- | --- | --- | --- | --- | --- | --- | --- | --- | --- |
| **KSS1** | Spring | 89 | Aphotic | 7.74^e^ ± 0.08 | | 8.39^a^ ± 0.13 | | 43.2^a^ ± 0.14 | | 11.36^c^ ± 0.22 | | 203^i^ ± 0.07 | | 353.8^e^ ± 0.69 | | 16.73^f^ ± 0.15 | |  |
| **KSS2** | Spring | 109 | Aphotic | 12.73^b^ ± 0.51 | | 7.84^bcd^ ± 0.31 | | 25.6^f^ ± 0.51 | | 12.78^b^ ± 0.21 | | 298^d^ ± 0.65 | | 356.24^d^ ± 0.04 | | 17.13^f^ ± 0.03 | |  |
| **KSS3** | Spring | 106 | Aphotic | 9.4^d^ ± 0.23 | | 6.57^fg^ ± 0.47 | | 43.2^a^ ± 0 | | 9.94^d^ ± 0.71 | | 332^c^ ± 0.85 | | 375.76^c^ ± 0.58 | | 15.12^h^ ± 0.07 | |  |
| **KSS4** | Spring | 176 | Aphotic | 4.98^g^ ± 0.17 | | 3.79^j^ ± 0.35 | | 33.6^b^ ± 0.24 | | 9.94^d^ ± 0.92 | | 269^e^ ± 0.83 | | 265.96^g^ ± 0.59 | | 17.73^e^ ± 0.58 | |  |
| **KSS5** | Spring | 189 | Aphotic | 1.29^l^ ± 0.28 | | 6.95^ef^ ± 0.04 | | 25.6^f^ ± 0.04 | | 11.36^c^ ± 0.34 | | 267^f^ ± 0.17 | | 219.6^j^ ± 0.41 | | 22.04^c^ ± 0 | |  |
| **KSS6** | Spring | 199 | Aphotic | 9.77^d^ ± 0.05 | | 5.96^h^ ± 0.02 | | 27.2^e^ ± 0.06 | | 11.36^c^ ± 0.15 | | 207^h^ ± 0.1 | | 261.08^h^ ± 0.03 | | 18.06^e^ ± 0.01 | |  |
| **KSS7** | Spring | 217 | Aphotic | 2.21^jk^ ± 0.17 | | 4.49^i^ ± 0.29 | | 28.8^d^ ± 0.32 | | 11.36^c^ ± 0.19 | | 206^h^ ± 0.85 | | 261.08^h^ ± 0.01 | | 21.68^c^ ± 0.4 | |  |
| **KSS8** | Spring | 235 | Aphotic | 1.6^kl^ ± 0.25 | | 8.15^abc^ ± 0.04 | | 19.2^h^ ± 0.1 | | 7.1^f^ ± 0.05 | | 156.5^j^ ± 0.31 | | 129.32^k^ ± 0.13 | | 10.02^i^ ± 0.01 | |  |
| **MPS1** | Spring | 300 | Aphotic | 0.01^m^ ± 0.01 | | 7.39^de^ ± 0.11 | | 16^i^ ± 0.68 | | 19.02^a^ ± 0.01 | | 93^l^ ± 0.95 | | 234.24^i^ ± 0.23 | | 16.05^g^ ± 0.03 | |  |
| **LBS1** | Drip | 125 | Aphotic | 2.39^ij^ ± 0.1 | | 8.48^a^ ± 0.29 | | 11.2^j^ ± 0.07 | | 8.52^e^ ± 0.48 | | 59.7^m^ ± 0.17 | | 87.84^m^ ± 0.1 | | 5.84^j^ ± 0.47 | |  |
| **LBS2** | Drip | 150 | Aphotic | 2.95^i^ ± 0.05 | | 7.65^cd^ ± 0.1 | | 4.8^l^ ± 0.2 | | 11.36^c^ ± 0.03 | | 12.5^o^ ± 0.26 | | 36.6^o^ ± 0.07 | | 1.26^k^ ± 0.2 | |  |
| **LBS3** | Pool | 250 | Aphotic | 21.95^a^ ± 0.86 | | 6.42^gh^ ± 0.19 | | 24^g^ ± 0.13 | | 8.52^e^ ± 0 | | 421^a^ ± 0.85 | | 500.2^a^ ± 0.1 | | 29.34^a^ ± 0.2 | |  |
| **LBS4** | Spring | 650 | Aphotic | 10.51^c^ ± 0.38 | | 8.1^abc^ ± 0.09 | | 27.2^e^ ± 0.1 | | 7.1^f^ ± 0.07 | | 361^b^ ± 0.8 | | 424.56^b^ ± 0.31 | | 23.41^b^ ± 0.39 | |  |
| **LBD1** | Drip | 715 | Aphotic | 5.9^f^ ± 0.02 | | 3.98^j^ ± 0.6 | | 6.4^k^ ± 0.19 | | 12.78^b^ ± 0.06 | | 58.4^n^ ± 0.17 | | 46.36^n^ ± 0.13 | | 5.59^j^ ± 0.21 | |  |
| **LBD2** | Drip | 825 | Aphotic | 3.87^h^ ± 0.37 | | 8.3^ab^ ± 0.24 | | 30.4^c^ ± 0.16 | | 9.94^d^ ± 0.23 | | 227^g^ ± 0.84 | | 287.92^f^ ± 0.46 | | 18.62^d^ ± 0.54 | |  |
| **LBD3** | Drip | 850 | Aphotic | 0.16^m^ ± 0.02 | | 8.33^ab^ ± 0.28 | | 11.2^j^ ± 0.15 | | 12.78^b^ ± 0.58 | | 109.4^k^ ± 0.33 | | 122^l^ ± 0.54 | | BDL | |  |

**Supplementary Table 1. Characteristics and geochemistry of water samples collected from Khasi hills caves, Meghalaya**

| **Sample ID** | **Potassium (mg/l)** | **Magnesium (mg/l)** | **Sodium (mg/l)** | **Nitrate (mg/l)** | **pH** | **Salinity (mg/l)** | **Total Carbon (mg/l)** | **Total Dissolved Solids** | **Total Hardness (mg/l)** | **Total Organic Carbon**  **(mg/l)** | |
| --- | --- | --- | --- | --- | --- | --- | --- | --- | --- | --- | --- |
| **KSS1** | 0.7^de^ ± 0.1 | 10.69^k^ ± 0.44 | 1.5^ef^ ± 0.12 | 3.33^cd^ ± 0.21 | 7.72^a^ ± 0.046 | 91.2^j^ ± 0.12 | 17.11^h^ ± 0.01 | 147^h^ ± 0.1 | 152^h^ ± 0 | 0.37^f^ ± 0.01 |  |
| **KSS2** | 0.7^de^ ± 0.14 | 25.27^f^ ± 0.15 | 1.6^ef^ ± 0.13 | 3.26^de^ ± 0.03 | 7.55^abcde^ ± 0.098 | 134^d^ ± 0.2 | 17.66^g^ ± 0.46 | 212^d^ ± 0.1 | 168^g^ ± 0.43 | 0.53^e^ ± 0 |  |
| **KSS3** | 0.7^de^ ± 0.1 | 7.78^l^ ± 0.03 | 1.6^ef^ ± 0.2 | 0.86^i^ ± 0.06 | 7.5^abcdef^ ± 0.1 | 149^c^ ± 0.03 | 15.13^j^ ± 0.1 | 236^c^ ± 0 | 140^i^ ± 0.84 | 0.002^h^ ± 0 |  |
| **KSS4** | 0.8^d^ ± 0.06 | 16.52^i^ ± 0.07 | 1.5^ef^ ± 0.26 | 2.66^f^ ± 0.06 | 7.43^bcdef^ ± 0.252 | 124^f^ ± 0.5 | 18.29^f^ ± 0.28 | 192^f^ ± 0.5 | 152^h^ ± 0.24 | 0.57^e^ ± 0.01 |  |
| **KSS5** | 1.2^c^ ± 0 | 32.07^e^ ± 0.03 | 1.7^e^ ± 0.09 | 1.93^g^ ± 0.13 | 7.6^abcd^ ± 0.18 | 130^e^ ± 0.17 | 23.05^c^ ± 0 | 193^e^ ± 0.18 | 196^e^ ± 0.25 | 1.01^b^ ± 0 |  |
| **KSS6** | 1.1^c^ ± 0.04 | 24.3^g^ ± 0.29 | 2.1^d^ ± 0.1 | 3.2^de^ ± 0.09 | 7.4^cdef^ ± 0.17 | 93.4^i^ ± 0.02 | 17.21^h^ ± 0.1 | 147^h^ ± 0.06 | 168^g^ ± 0.31 | 0.85^c^ ± 0.01 |  |
| **KSS7** | 1.6^b^ ± 0.1 | 37.9^c^ ± 0.28 | 2.2^d^ ± 0.14 | 2.46^f^ ± 0.04 | 7.3^efg^ ± 0.13 | 95.5^h^ ± 0.48 | 21.94^d^ ± 0.04 | 145^i^ ± 0.22 | 228^c^ ± 0 | 0.85^c^ ± 0.01 |  |
| **KSS8** | 1.1^c^ ± 0.06 | 98.1^a^ ± 0.02 | 1.4^f^ ± 0.27 | 4.4^b^ ± 0.03 | 7.7^ab^ ± 0.29 | 72.5^k^ ± 0.36 | 11.52^k^ ± 0.16 | 112^j^ ± 0.1 | 452^a^ ± 0.1 | 1.5^a^ ± 0.1 |  |
| **MPS1** | 0.633^e^ ± 0.05 | 32.07^e^ ± 0.07 | 3^b^ ± 0.1 | 1.4^h^ ± 0.19 | 7.3^efg^ ± 0 | 43.5^m^ ± 0.25 | 16.58^i^ ± 0.14 | 65.3^l^ ± 0.05 | 172^f^ ± 0.2 | 0.53^e^ ± 0.03 |  |
| **LBS1** | 0.3^f^ ± 0 | 11.66^j^ ± 0.21 | 1.6^ef^ ± 0.11 | BDL | 7.3^efg^ ± 0.07 | 29.2^n^ ± 0.01 | 6.87^l^ ± 0.03 | 42.3^m^ ± 0.3 | 76^l^ ± 0.22 | 1.03^b^ ± 0.03 |  |
| **LBS2** | 0.2^fg^ ± 0.01 | 7.77^l^ ± 0.07 | 1.3^f^ ± 0.1 | BDL | 7.2^fg^ ± 0.18 | 11.3^p^ ± 0.1 | 1.96^m^ ± 0.24 | 18.9^o^ ± 0.02 | 44^m^ ± 0.29 | 0.69^d^ ± 0 |  |
| **LBS3** | 1.9^a^ ± 0.01 | 47.62^b^ ± 0.34 | 6.2^a^ ± 0 | 3.5^c^ ± 0.2 | 7.1^g^ ± 0.03 | 189^a^ ± 0.69 | 29.6^a^ ± 0.18 | 299^a^ ± 0.12 | 256^b^ ± 0 | 0.26^g^ ± 0.03 |  |
| **LBS4** | 1.7^b^ ± 0.1 | 34.02^d^ ± 0 | 6^a^ ± 0.3 | 3.06^e^ ± 0 | 7.7^abc^ ± 0.01 | 162^b^ ± 0.38 | 24.08^b^ ± 0.08 | 256^b^ ± 0.4 | 208^d^ ± 0.3 | 0.67^d^ ± 0.01 |  |
| **LBD1** | 0.116^g^ ± 0.029 | 37.9^c^ ± 0.2 | 1.8^e^ ± 0.11 | 0.06^j^ ± 0.02 | 7.5^abcdef^ ± 0.01 | 28.2^o^ ± 0.18 | 6.57^l^ ± 0.04 | 40.3^n^ ± 0.29 | 172^f^ ± 0.35 | 0.98^b^ ± 0.04 |  |
| **LBD2** | 0.8^d^ ± 0.1 | 11.66^j^ ± 0.13 | 2.5^c^ ± 0.22 | 4.6^a^ ± 0.2 | 7.4^def^ ± 0.1 | 102^g^ ± 0 | 18.99^e^ ± 0.05 | 162^g^ ± 0 | 124^j^ ± 0.15 | 0.37^f^ ± 0.01 |  |
| **LBD3** | 0.8^d^ ± 0.15 | 21.38^h^ ± 0.24 | 1.7^e^ ± 0.1 | 1.46^h^ ± 0.06 | 7.6^abcd^ ± 0.3 | 50.5^l^ ± 0.47 | BDL | 77.7^k^ ± 0.02 | 116^k^ ± 0.09 | BDL |  |

| - BDL: Below Detection Level |
| --- |
| - (ANOVA and Duncuns Multiple range test performed) |
| - All data represent mean of triplicate (±) SD. Means followed by different letters are significantly different at the 0.05 probability level, grouped into classes   a, b, c, d, e, f, g, h, i, j, k, l, m, n, o, p. |
