# Supplementary material for: Cultivable microbial diversity in speleothems using MALDI-TOF spectrometry and DNA sequencing from Krem Soitan, Krem Lawbah, Krem Mawpun, Khasi Hills, Meghalaya, India
Source: Arch Microbiol. 2022 Jul 17;204(8):495. doi: 10.1007/s00203-022-02916-8 (PMC9288962; doi:10.1007/s00203-022-02916-8)
Supplement: Supplementary file 7 — Supplementary file7 (DOCX 17 KB) [file 203_2022_2916_MOESM7_ESM.docx]

| **Supplementary Table 2- Summary of nucleotide sequences accession numbers** |
| --- |

| **Sequence** | **Name** | **Accession number** | **Id for Sequence deposition** | **Strain ID** | **Sample Name** |
| --- | --- | --- | --- | --- | --- |
| Seq2 | *Arthrobacter oxydans* | MG733189 | KSSMR02 | 1a_R2A_15 | KSSM1 |
| Seq3 | *Arthrobacter oryzae* | MG733190 | KSSMR03 | 1a_R2A_18 | KSSM1 |
| Seq4 | *Pseudomonas chlororphis* | MG733191 | KSSMR04 | 1b_R2A_02 | KSSM1 |
| Seq5 | *Pseudarthrobacter oxydans* | MG733192 | KSSMR05 | 1b_R2A_03 | KSSM1 |
| Seq6 | *Bacillus altitudinis* | MG733193 | KSSMR06 | 1b_R2A_12 | KSSM1 |
| Seq7 | *Pseudomonas koreensis* | MG733194 | KSSMR07 | 1b_R2A_14 | KSSM1 |
| Seq11 | *Pseudomonas granadensis* | MG733198 | KSSTR11 | 03_R2A_03 | KSST2 |
| Seq12 | *Pseudomonas granadensis* | MG733199 | KSSTR12 | 03_R2A_10 | KSST2 |
| Seq13 | *Pseudomonas granadensis* | MG733200 | KSSTR13 | 03_R2A_11 | KSST2 |
| Seq14 | *Pseudomonas alkylphenolica* | MG733201 | KSSTR14 | 04_R2A_04 | KSST3 |
| Seq15 | *Paenarthrobacter nicotinovorans* | MG733202 | KSSTR15 | 04_R2A_08 | KSST3 |
| Seq16 | *Paenarthrobacter nicotinovorans* | MG733203 | KSSTR16 | 04_R2A_10 | KSST3 |
| Seq17 | *Pseudomonas alkylphenolica* | MG733204 | KSSTR17 | 04_R2A_13 | KSST3 |
| Seq18 | *Pseudomonas alkylphenolica* | MG733205 | KSSTR18 | 04_R2A_21 | KSST3 |
| Seq19 | *Enterobacter cloacae* | MG733206 | KSSTM19 | 4A_M9_03 | KSST3 |
| Seq20 | *Enterobacter ludwigii* | MG733207 | KSSTM20 | 4A_M9_06 | KSST3 |
| Seq21 | *Enterobacter tabaci* | MG733208 | KSSTM22 | 4A_M9_19 | KSST3 |
| Seq22 | *Enterobacter tabaci* | MG733209 | KSSTM23 | 4B_M9_03 | KSST3 |
| Seq23 | *Enterobacter cloacae* | MG733210 | KSSTM24 | 4B_M9_12 | KSST3 |
| Seq24 | *Pseudarthrobacter oxydans* | MG733211 | KSSTR25 | 05_R2A_01 | KSST4 |
| Seq25 | *Pseudarthrobacter oxydans* | MG733212 | KSSTR26 | 05_R2A_02 | KSST4 |
| Seq26 | *Pseudarthrobacter oxydans* | MG733213 | KSSTR27 | 05_R2A_09 | KSST4 |
| Seq27 | *Pseudarthrobacter oxydans* | MG733214 | KSSTR28 | 05_R2A_10 | KSST4 |
| Seq28 | *Deinococcus ficus* | MG733215 | KSSTR29 | 05_R2A_13 | KSST4 |
| Seq29 | *Arthrobacter ginsengisoli* | MG733216 | KSSTR30 | 05_R2A_16 | KSST4 |
| Seq30 | *Pseudarthrobacter oxydans* | MG733217 | KSSTR31 | 05_R2A_18 | KSST4 |
| Seq31 | *Enterobacter asburiae* | MG733218 | KSSTM32 | 06A_M9_12 | KSST5 |
| Seq32 | *Pseudomonas alkylphenolica* | MG733219 | KSSTR33 | 06A_R2A_01 | KSST5 |
| Seq33 | *Paenarthrobacter nicotinovorans* | MG733220 | KSSTR34 | 06A_R2A_06 | KSST5 |
| Seq34 | *Arthrobacter oryzae* | MG733221 | KSSTR35 | 06A_R2A_08 | KSST5 |
| Seq35 | *Paenarthrobacter nicotinovorans* | MG733222 | KSSTR37 | 06A_R2A_14 | KSST5 |
| Seq36 | *Paenarthrobacter nicotinovorans* | MG733223 | KSSTR38 | 06A_R2A_16 | KSST5 |
| Seq37 | *Arthrobacter methylotrophus* | MG733224 | KSSTR39 | 06B_R2A_06 | KSST5 |
| Seq38 | *Pseudomonas alkylphenolica* | MG733225 | KSSTR40 | 06B_R2A_01 | KSST5 |
| Seq39 | *Pseudomonas alkylphenolica* | MG733226 | KSSTR41 | 06B_R2A_04 | KSST5 |
| Seq40 | *Microbacterium oxydans* | MG733227 | KSSTR42 | 06B_R2A_09 | KSST5 |
| Seq41 | *Arthrobacter methylotrophus* | MG733228 | KSSTR43 | 06B_R2A_20 | KSST5 |
| Seq42 | *Pseudomonas guariconensis* | MG733229 | KSSTM44 | 08_M9_08 | KSST7 |
| Seq43 | *Enterobacter cloacae* | MG733230 | KSSTM45 | 08_M9_17 | KSST7 |
| Seq44 | *Enterobacter tabaci* | MG733231 | KSSTM46 | 08_M9_20 | KSST7 |
| Seq45 | *Paenibacillus polymyxa* | MG733232 | KSSTR47 | 08_R2A_01 | KSST7 |
| Seq46 | *Pseudomonas alkylphenolica* | MG733233 | KSSTR48 | 08_R2A_05 | KSST7 |
| Seq47 | *Pseudomonas alkylphenolica* | MG733234 | KSSTR49 | 08_R2A_11 | KSST7 |
| Seq48 | *Pseudomonas alkylphenolica* | MG733235 | KSSTR50 | 08_R2A_12 | KSST7 |
| Seq49 | *Pseudomonas granadensis* | MG733236 | KSSTR51 | 08_R2A_14 | KSST7 |
| Seq50 | *Pseudomonas donghuensis* | MG733237 | KSSTR53 | 08_R2A_20 | KSST7 |
| Seq52 | *Enterobacter cloacae* | MG733239 | KSSTM55 | 09_M9_08 | KSST8 |
| Seq53 | *Flavobacterium tructae* | MG733240 | KSSTR56 | 9_R2A_11 | KSST8 |
| Seq54 | *Flavobacterium hercynium* | MG733241 | KSSTR57 | 9_R2A_13 | KSST8 |
| Seq55 | *Pseudomonas nitroreducens* | MG733242 | KSSTR58 | 9_R2A_19 | KSST8 |
| Seq57 | *Kocuria rhizophila* | MG733244 | LBSTR61 | 12_R2A_01 | LBST2 |
| Seq58 | *Bacillus safensis* | MG733245 | LBSTR64 | 13_R2A_09 | LBST3 |
| Seq59 | *Arthrobacter pascens* | MG733246 | LBSTR65 | 13_R2A_12 | LBST3 |
| Seq60 | *Pseudarthrobacter polychromogenes* | MG733247 | LBSTR66 | 13_R2A_15 | LBST3 |
| Seq61 | *Pseudarthrobacter polychromogenes* | MG733248 | LBSTR67 | 13B_R2A_02 | LBST3 |
| Seq62 | *Arthrobacter oxydans* | MG733249 | LBSTR68 | 13B_R2A_04 | LBST3 |
| Seq63 | *Pseudarthrobacter polychromogenes* | MG733250 | LBSTR69 | 13B_R2A_05 | LBST3 |
| Seq64 | *Streptococcus gallolyticus* | MG733251 | LBSTR70 | 13B_R2A_12 | LBST3 |
| Seq65 | *Arthrobacter oxydans* | MG733252 | LBSTR71 | 13B_R2A_14 | LBST3 |
| Seq66 | *Pseudarthrobacter polychromogenes* | MG733253 | LBSTR72 | 13B_R2A_19 | LBST3 |
| Seq67 | *Arthrobacter ginsengisoli* | MG733254 | LBSTR73 | 13B_R2A_20 | LBST3 |
| Seq68 | *Paenarthrobacter nicotinovorans* | MG733255 | LBWDR74 | 14_R2A_01 | LBWD1 |
| Seq82 | *Arthrobacter oxydans* | MG733269 | LBWDR90 | 16_R2A_07 | LBWD3 |
| Seq83 | *Pseudarthrobacter polychromogenes* | MG733270 | LBWDR91 | 16_R2A_10 | LBWD3 |
| Seq84 | *Staphylococcus warneri* | MG733271 | LBWDR93 | 17_R2A_07 | LBWD4 |
| Seq85 | *Arthrobacter ginsengisoli* | MG733272 | LBWDR94 | 17_R2A_14 | LBWD4 |
| Seq86 | *Pseudomonas koreensis* | MG733273 | LBWDR95 | 17B_R2A_08 | LBWD4 |
| Seq87 | *Pseudomonas granadensis* | MG733274 | LBWDR96 | 17B_R2A_13 | LBWD4 |
