# Supplementary material for: Cultivable microbial diversity in speleothems using MALDI-TOF spectrometry and DNA sequencing from Krem Soitan, Krem Lawbah, Krem Mawpun, Khasi Hills, Meghalaya, India
Source: Arch Microbiol. 2022 Jul 17;204(8):495. doi: 10.1007/s00203-022-02916-8 (PMC9288962; doi:10.1007/s00203-022-02916-8)
Supplement: Supplementary file 8 — Supplementary file8 (DOCX 14 KB) [file 203_2022_2916_MOESM8_ESM.docx]

**Supplementary Table 3- Phylum wise distribution of major phylogenetic groups of bacteria**

| **Sample Id**  **Phylum** | **KSSMc1** | **KSSTc2** | **KSSTc3** | **KSSTc4** | **KSSTc5** | **KSSTc7** | **KSSTc8** | **MPSTc1** | **LBSTc1** | **LBSTc2** | **LBSTc3** | **LBWDc1** | **LBWDc2** | **LBWDc3 LBWDc4** |
| --- | --- | --- | --- | --- | --- | --- | --- | --- | --- | --- | --- | --- | --- | --- |
| **Actinobacteria** | 7 | 2 | 5 | 7 | 22 | 0 | 0 | 11 | 0 | 1 | 19 | 1 | 0 | 9 5 |
| **Alphaproteobacteria** |  |  | 1 |  |  |  |  |  |  |  |  |  |  | 2 |
| **Bacteroidetes** |  |  |  |  |  |  | 2 |  |  |  |  |  |  |  |
| **Betaproteobacteria** | 1 |  |  |  |  |  |  |  |  |  |  |  |  |  |
| **Deinococcus-Thermus** |  |  |  | 1 |  |  |  |  |  |  |  |  |  |  |
| **Firmicutes** | 1 | 0 | 0 | 0 | 0 | 1 | 0 | 0 | 1 | 0 | 2 | 0 | 0 | 0 17 |
| **Gammaproteobacteria** | 38 | 21 | 18 | 0 | 15 | 26 | 25 | 0 | 0 | 0 | 3 | 0 | 31 | 0 |
