# Supplementary material for: Cultivable microbial diversity in speleothems using MALDI-TOF spectrometry and DNA sequencing from Krem Soitan, Krem Lawbah, Krem Mawpun, Khasi Hills, Meghalaya, India
Source: Arch Microbiol. 2022 Jul 17;204(8):495. doi: 10.1007/s00203-022-02916-8 (PMC9288962; doi:10.1007/s00203-022-02916-8)
Supplement: Supplementary file 9 — Supplementary file9 (DOCX 14 KB) [file 203_2022_2916_MOESM9_ESM.docx]

|  | **KSSMc1** | **KSSTc1** | **KSSTc2** | **KSSTc3** | **KSSTc4** | **KSSTc5** | **KSSTc6** | **KSSTc7** | **KSSTc8** | **MPSTc1** | **LBSTc1** | **LBSTc2** | **LBSTc3** | **LBWDc1** | **LBWDc2** | **LBWDc3** | **LBWDc4** |
| --- | --- | --- | --- | --- | --- | --- | --- | --- | --- | --- | --- | --- | --- | --- | --- | --- | --- |
| *Arthrobacter* | 6 | 0 | 2 | 3 | 2 | 18 | 0 |  |  | 11 |  |  | 15 |  |  | 8 | 5 |
| *Bacillus* | 1 | 0 |  |  |  |  | 0 |  |  |  |  |  | 1 |  |  |  |  |
| *Enterobacter* |  | 0 |  | 5 |  | 1 | 0 | 2 | 1 |  |  |  |  |  |  |  |  |
| *Pseudarthrobacter* | 1 | 0 |  |  | 5 |  | 0 |  |  |  |  |  | 4 |  |  | 1 |  |
| *Pseudomonas* | 38 | 0 | 21 | 13 |  | 12 | 0 | 21 | 22 |  |  |  | 3 |  | 31 |  | 2 |
| *Staphylococcus* |  | 0 |  |  |  |  | 0 |  |  |  | 1 |  |  |  |  |  | 17 |
| *Variovorax* | 1 | 0 |  |  |  |  | 0 |  |  |  |  |  |  |  |  |  |  |
| *Kocuria* |  | 0 |  |  |  |  | 0 |  |  |  |  | 1 |  |  |  |  |  |
| *Microbacterium* |  | 0 |  |  |  | 1 | 0 |  |  |  |  |  |  |  |  |  |  |
| *Brevundimonas* |  | 0 |  | 1 |  |  | 0 |  |  |  |  |  |  |  |  |  |  |
| *Deinococcus* |  | 0 |  |  | 1 |  | 0 |  |  |  |  |  |  |  |  |  |  |
| *Paenarthrobacter* |  | 0 |  | 2 |  | 3 | 0 |  |  |  |  |  |  | 1 |  |  |  |
| *Acinetobacter* |  | 0 |  |  |  | 2 | 0 | 3 | 2 |  |  |  |  |  |  |  |  |
| *Paenibacillus* |  | 0 |  |  |  |  | 0 | 1 |  |  |  |  |  |  |  |  |  |
| *Flavobacterium* |  | 0 |  |  |  |  | 0 |  | 2 |  |  |  |  |  |  |  |  |
| *Streptococcus* |  | 0 |  |  |  |  | 0 |  |  |  |  |  | 1 |  |  |  |  |

**Supplementary Table 4- Genus wise distribution of major phylogenetic groups of bacteria**
