# Supplementary material for: Cultivable microbial diversity in speleothems using MALDI-TOF spectrometry and DNA sequencing from Krem Soitan, Krem Lawbah, Krem Mawpun, Khasi Hills, Meghalaya, India
Source: Arch Microbiol. 2022 Jul 17;204(8):495. doi: 10.1007/s00203-022-02916-8 (PMC9288962; doi:10.1007/s00203-022-02916-8)
Supplement: Supplementary file 10 — Supplementary file10 (DOCX 24 KB) [file 203_2022_2916_MOESM10_ESM.docx]

**Supplementary Table 5- Species wise distribution of major phylogenetic groups of bacteria**

|  | **KS**  **SMc1** | **KS**  **STc1** | | **KS**  **STc2** | | **KS**  **STc3** | | **KS**  **STc4** | | **KS**  **STc5** | | **KS**  **STc6** | | **KS**  **STc7** | | **KS**  **STc8** | | **MP**  **STc1** | | **LB**  **STc1** | | **LB**  **STc2** | | **LB**  **STc3** | | **LB**  **WDc1** | | **LB**  **WDc2** | | **LB**  **WDc3** | | **LB**  **WDc4** | |  |
| --- | --- | --- | --- | --- | --- | --- | --- | --- | --- | --- | --- | --- | --- | --- | --- | --- | --- | --- | --- | --- | --- | --- | --- | --- | --- | --- | --- | --- | --- | --- | --- | --- | --- | --- |
| *Acinetobacter johnsonii* |  |  | |  | |  | |  | | 2 | |  | |  | | 2 | |  | |  | |  | |  | |  | |  | |  | |  | |  |
| *Acinetobacter lwoffii* |  |  | |  | |  | |  | |  | |  | | 3 | |  | |  | |  | |  | |  | |  | |  | |  | |  | |  |
| *Arthrobacter aurescens* | 1 |  | |  | |  | |  | | 1 | |  | |  | |  | |  | |  | |  | |  | |  | |  | |  | |  | |  |
| *Arthrobacter ginsengisoli* |  |  | |  | |  | | 1 | |  | |  | |  | |  | |  | |  | |  | | 1 | |  | |  | |  | | 1 | |  |
| *Arthrobacter histidinolovorans* |  |  | |  | |  | |  | | 5 | |  | |  | |  | |  | |  | |  | |  | |  | |  | |  | |  | |  |
| *Arthrobacter ilicis* |  |  | |  | |  | |  | | 1 | |  | |  | |  | |  | |  | |  | |  | |  | |  | |  | |  | |  |
| *Arthrobacter methylotrophus* |  |  | |  | |  | |  | | 2 | |  | |  | |  | |  | |  | |  | |  | |  | |  | |  | |  | |  |
| *Arthrobacter nicotinovorans* |  |  | |  | |  | |  | | 7 | |  | |  | |  | |  | |  | |  | |  | |  | |  | |  | |  | |  |
| *Arthrobacter oryzae* | 1 |  | |  | |  | |  | | 1 | |  | |  | |  | |  | |  | |  | |  | |  | |  | |  | |  | |  |
| *Arthrobacter oxydans* | 3 |  | | 1 | |  | |  | |  | |  | |  | |  | | 7 | |  | |  | | 5 | |  | |  | | 4 | | 1 | |  |
| *Arthrobacter pascens* |  |  | |  | |  | |  | |  | |  | |  | |  | |  | |  | |  | | 2 | |  | |  | |  | |  | |  |
| *Arthrobacter polychromogenes* |  |  | |  | | 2 | | 1 | |  | |  | |  | |  | | 2 | |  | |  | | 6 | |  | |  | | 2 | | 1 | |  |
| *Arthrobacter sulfonivorans* | 1 |  | | 1 | | 1 | |  | | 1 | |  | |  | |  | | 2 | |  | |  | | 1 | |  | |  | | 2 | | 2 | |  |
| *Bacillus altitudinis* | 1 |  | |  | |  | |  | |  | |  | |  | |  | |  | |  | |  | |  | |  | |  | |  | |  | |  |
| *Bacillus safensi* |  |  | |  | |  | |  | |  | |  | |  | |  | |  | |  | |  | | 1 | |  | |  | |  | |  | |  |
| *Brevundimonas vesicularis* |  |  | |  | | 1 | |  | |  | |  | |  | |  | |  | |  | |  | |  | |  | |  | |  | |  | |  |
| *Deinococcus ficus* |  |  | |  | |  | | 1 | |  | |  | |  | |  | |  | |  | |  | |  | |  | |  | |  | |  | |  |
| *Enterobacter asburiae* |  |  | |  | |  | |  | | 1 | |  | |  | |  | |  | |  | |  | |  | |  | |  | |  | |  | |  |
| *Enterobacter cloacae* |  |  | |  | | 2 | |  | |  | |  | | 1 | | 1 | |  | |  | |  | |  | |  | |  | |  | |  | |  |
| *Enterobacter ludwigii* |  |  | |  | | 1 | |  | |  | |  | |  | |  | |  | |  | |  | |  | |  | |  | |  | |  | |  |
| *Enterobacter tabaci* |  |  | |  | | 2 | |  | |  | |  | | 1 | |  | |  | |  | |  | |  | |  | |  | |  | |  | |  |
| *Flavobacterium hercynium* |  |  | |  | |  | |  | |  | |  | |  | | 1 | |  | |  | |  | |  | |  | |  | |  | |  | |  |
| *Flavobacterium tructae* |  |  | |  | |  | |  | |  | |  | |  | | 1 | |  | |  | |  | |  | |  | |  | |  | |  | |  |
| *Kocuria rhizophila* |  |  | |  | |  | |  | |  | |  | |  | |  | |  | |  | | 1 | |  | |  | |  | |  | |  | |  |
| *Microbacterium oxydans* |  |  | |  | |  | |  | | 1 | |  | |  | |  | |  | |  | |  | |  | |  | |  | |  | |  | |  |
| *Paenarthrobacter nicotinovorans* |  |  | |  | | 2 | |  | | 3 | |  | |  | |  | |  | |  | |  | |  | | 1 | |  | |  | |  | |  |
| *Paenibacillus polymyxa* |  |  | |  | |  | |  | |  | |  | | 1 | |  | |  | |  | |  | |  | |  | |  | |  | |  | |  |
| *Pseudarthrobacter oxydans* | 1 |  | |  | |  | | 5 | |  | |  | |  | |  | |  | |  | |  | |  | |  | |  | |  | |  | |  |
| *Pseudarthrobacter polychromogenes* | |  | |  | |  | |  | |  | |  | |  | |  | |  | |  | |  | | 4 | |  | |  | | 1 | |  | |  |
| *Pseudomonas aeruginosa* |  |  | |  | |  | |  | |  | |  | |  | | 1 | |  | |  | |  | |  | |  | |  | |  | |  | |  |
| *Pseudomonas alkylphenolica* |  |  | |  | | 3 | |  | | 3 | |  | | 3 | |  | |  | |  | |  | |  | |  | |  | |  | |  | |  |
| *Pseudomonas asplenii* |  |  | |  | |  | |  | | 1 | |  | |  | |  | |  | |  | |  | |  | |  | |  | |  | |  | |  |
| *Pseudomonas azotoformans* |  |  | |  | |  | |  | |  | |  | |  | | 1 | |  | |  | |  | |  | |  | | 1 | |  | |  | |  |
| *Pseudomonas brassicacearum* | 1 |  | | 2 | |  | |  | |  | |  | | 1 | |  | |  | |  | |  | |  | |  | |  | |  | |  | |  |
| *Pseudomonas brenneri* | 1 |  | |  | |  | |  | |  | |  | |  | |  | |  | |  | |  | |  | |  | |  | |  | |  | |  |
| *Pseudomonas caricapapayae* |  |  | |  | |  | |  | | 1 | |  | |  | |  | |  | |  | |  | |  | |  | |  | |  | |  | |  |
| *Pseudomonas chlororaphis* | 24 |  | | 2 | |  | |  | | 1 | |  | | 3 | |  | |  | |  | |  | | 1 | |  | | 11 | |  | |  | |  |
| *Pseudomonas cichorii* |  |  | |  | |  | |  | | 1 | |  | | 1 | | 2 | |  | |  | |  | |  | |  | |  | |  | |  | |  |
| *Pseudomonas congelans* |  |  | |  | | 1 | |  | |  | |  | | 1 | | 1 | |  | |  | |  | |  | |  | |  | |  | |  | |  |
| *Pseudomonas corrugata* | 2 |  | | 1 | |  | |  | | 1 | |  | | 4 | | 4 | |  | |  | |  | |  | |  | |  | |  | |  | |  |
| *Pseudomonas donghuensis* |  |  | |  | |  | |  | |  | |  | | 1 | |  | |  | |  | |  | |  | |  | |  | |  | |  | |  |
| *Pseudomonas extremorientalis* | 2 |  | | 1 | |  | |  | |  | |  | |  | |  | |  | |  | |  | |  | |  | |  | |  | |  | |  |
| *Pseudomonas fuscovaginae* | 1 |  | |  | |  | |  | |  | |  | |  | | 1 | |  | |  | |  | |  | |  | |  | |  | |  | |  |
| *Pseudomonas graminis* |  |  | |  | |  | |  | |  | |  | | 2 | |  | |  | |  | |  | |  | |  | |  | |  | |  | |  |
| *Pseudomonas granadensis* |  |  | | 3 | |  | |  | |  | |  | | 1 | |  | |  | |  | |  | |  | |  | |  | |  | | 1 | |  |
| *Pseudomonas grimontii* |  |  | |  | |  | |  | | 1 | |  | |  | |  | |  | |  | |  | |  | |  | |  | |  | |  | |  |
| *Pseudomonas guariconensis* |  |  | |  | |  | |  | |  | |  | | 1 | |  | |  | |  | |  | |  | |  | |  | |  | |  | |  |
| *Pseudomonas jessenii* |  |  | | 1 | |  | |  | |  | |  | |  | | 1 | |  | |  | |  | | 1 | |  | | 6 | |  | |  | |  |
| *Pseudomonas kilonensis* | 1 |  | |  | |  | |  | |  | |  | |  | |  | |  | |  | |  | |  | |  | |  | |  | |  | |  |
| *Pseudomonas koreensis* | 1 |  | | 3 | | 3 | |  | |  | |  | |  | | 1 | |  | |  | |  | |  | |  | | 3 | |  | | 1 | |  |
| *Pseudomonas lutea* | 1 |  | |  | |  | |  | | 1 | |  | | 3 | |  | |  | |  | |  | |  | |  | |  | |  | |  | |  |
| *Pseudomonas marginalis* |  |  | |  | | 1 | |  | |  | |  | |  | |  | |  | |  | |  | |  | |  | |  | |  | |  | |  |
| *Pseudomonas monteilii* | 1 |  | |  | |  | |  | |  | |  | |  | |  | |  | |  | |  | |  | |  | | 1 | |  | |  | |  |
| *Pseudomonas mosselii* |  |  | |  | | 2 | |  | |  | |  | |  | | 1 | |  | |  | |  | |  | |  | |  | |  | |  | |  |
| *Pseudomonas nitroreducens* |  |  | |  | |  | |  | |  | |  | |  | | 4 | |  | |  | |  | |  | |  | |  | |  | |  | |  |
| *Pseudomonas oleovorans* |  |  | |  | |  | |  | | 1 | |  | |  | |  | |  | |  | |  | |  | |  | |  | |  | |  | |  |
| *Pseudomonas poae* |  |  | | 1 | |  | |  | |  | |  | |  | |  | |  | |  | |  | |  | |  | |  | |  | |  | |  |
| *Pseudomonas proteolytica* |  |  | |  | |  | |  | |  | |  | |  | |  | |  | |  | |  | |  | |  | | 1 | |  | |  | |  |
| *Pseudomonas pseudoalcaligenes* |  |  | |  | |  | |  | | 1 | |  | |  | |  | |  | |  | |  | |  | |  | |  | |  | |  | |  |
| *Pseudomonas putida* |  |  | | 1 | | 3 | |  | |  | |  | |  | |  | |  | |  | |  | |  | |  | |  | |  | |  | |  |
| *Pseudomonas rhodesiae* |  |  | |  | |  | |  | |  | |  | |  | | 2 | |  | |  | |  | |  | |  | | 1 | |  | |  | |  |
| *Pseudomonas* sp. |  |  | |  | |  | |  | |  | |  | |  | | 1 | |  | |  | |  | |  | |  | |  | |  | |  | |  |
| *Pseudomonas thivervalensis* |  |  | | 1 | |  | |  | |  | |  | |  | | 1 | |  | |  | |  | |  | |  | | 1 | |  | |  | |  |
| *Pseudomonas tolaasii* |  |  | | 1 | |  | |  | |  | |  | |  | |  | |  | |  | |  | |  | |  | |  | |  | |  | |  |
| *Pseudomonas trivialis* |  |  | | 2 | |  | |  | |  | |  | |  | |  | |  | |  | |  | |  | |  | | 1 | |  | |  | |  |
| *Pseudomonas umsongensis* | 2 |  | | 2 | |  | |  | |  | |  | |  | | 1 | |  | |  | |  | | 1 | |  | | 4 | |  | |  | |  |
| *Pseudomonas vancouverensis* | 1 |  | |  | |  | |  | |  | |  | |  | |  | |  | |  | |  | |  | |  | | 1 | |  | |  | |  |
| *Staphylococcus hominis* |  |  | |  | |  | |  | |  | |  | |  | |  | |  | | 1 | |  | |  | |  | |  | |  | |  | |  |
| *Staphylococcus warneri* |  |  | |  | |  | |  | |  | |  | |  | |  | |  | |  | |  | |  | |  | |  | |  | | 17 | |  |
| *Streptococcus gallolyticus* |  |  | |  | |  | |  | |  | |  | |  | |  | |  | |  | |  | | 1 | |  | |  | |  | |  | |  |
| *Variovorax paradoxus* | 1 | |  | |  | |  | |  | |  | |  | |  | |  | |  | |  | |  | |  | |  | |  | |  | |  | |
