# Supplementary material for: Cultivable microbial diversity in speleothems using MALDI-TOF spectrometry and DNA sequencing from Krem Soitan, Krem Lawbah, Krem Mawpun, Khasi Hills, Meghalaya, India
Source: Arch Microbiol. 2022 Jul 17;204(8):495. doi: 10.1007/s00203-022-02916-8 (PMC9288962; doi:10.1007/s00203-022-02916-8)
Supplement: Supplementary file 11 — Supplementary file11 (DOCX 13 KB) [file 203_2022_2916_MOESM11_ESM.docx]

| **Sample Id** | **NCBI Match Organisms** | **Accession id** | **Query Coverage (%)** | **Similarity (%)** |
| --- | --- | --- | --- | --- |
| LBWDR93 | *Staphylococcus warneri* | MG733277.1 | 99 | 85.05 |
| LBSTR70 | [*Streptococcus gallolyticus*](https://blast.ncbi.nlm.nih.gov/Blast.cgi#alnHdr_1730310743) | MG733251.1 | 100 | 97.65 |
| KSSTR47 | [*Paenibacillus polymyx*](https://blast.ncbi.nlm.nih.gov/Blast.cgi#alnHdr_723613681) | MG733232.1 | 99 | 95.68 |
| KSSTR56 | [*Flavobacterium* sp. 142O](https://blast.ncbi.nlm.nih.gov/Blast.cgi#alnHdr_686478401) | MG733240.1 | 100 | 100 |
| KSSTR57 | *Flavobacterium tructae* | MG733241.1 | 100 | 99.09 |
| KSSTR39 | *Arthrobacter* sp. | MG733224.1 | 100 | 99.15 |
| KSSTR43 | [*Arthrobacter methylotrophus*](https://blast.ncbi.nlm.nih.gov/Blast.cgi#alnHdr_1317469158) | MG733228.1 | 100 | 95.69 |
| KSSMR04 | [*Pseudomonas* sp.](https://blast.ncbi.nlm.nih.gov/Blast.cgi#alnHdr_60687385) | MG733191.1 | 100 | 97.15 |
| KSSTM44 | [*Pseudomonas* sp.](https://blast.ncbi.nlm.nih.gov/Blast.cgi#alnHdr_686478432) | MG733229.1 | 100 | 98.68 |
| KSSTM46 | [*Enterobacter cloacae*](https://blast.ncbi.nlm.nih.gov/Blast.cgi#alnHdr_1317469173) | MG733231.1 | 98 | 92.03 |

**Supplementary Table 6. NCBI Blast match of specific sequences which formed separate clade in phylogenetic tree**
