# Supplementary material for: Cultivable microbial diversity in speleothems using MALDI-TOF spectrometry and DNA sequencing from Krem Soitan, Krem Lawbah, Krem Mawpun, Khasi Hills, Meghalaya, India
Source: Arch Microbiol. 2022 Jul 17;204(8):495. doi: 10.1007/s00203-022-02916-8 (PMC9288962; doi:10.1007/s00203-022-02916-8)
Supplement: Supplementary file 12 — Supplementary file12 (DOCX 16 KB) [file 203_2022_2916_MOESM12_ESM.docx]

**Supplementary Table 7. NCBI Blast match of specific sequences from wall scrapings**

| Sample Id/ Query sequence | NCBI Match Organisms | Accession Id | Similarity (%) | Isolated From |
| --- | --- | --- | --- | --- |
| LBWDR74 | *Kocuria* sp. L5 | DQ192212.1 | 95.29% | (Kartchner Caverns, USA)  Culturable microbial diversity and the impact of tourism in Kartchner Caverns, Arizona |
| LBWDR90 | *Arthrobacter sulfonivorans* | FR669674.1 | 99.49% | (Cave sediment: Herrenberg Cave, Germany) Calcite mineralization by karstic cave bacteria |
| LBWDR94 | *Arthrobacter sulfonivorans* | FR669674.1 | 99.15% | (Cave sediment: Herrenberg Cave, Germany) Calcite mineralization by karstic cave bacteria |
| LBWDR93 | *Staphylococcus equorum subsp. equorum strain ML5-8* | JX144958.1 | 83.66% | (Mawmluh cave, Meghalaya, India)  Biofilm bacteria from Mawmluh caves of India |
